# Clade-Specific Quantitative Analysis of Photosynthetic Gene Expression in *Prochlorococcus*

**DOI:** 10.1371/journal.pone.0133207

**Published:** 2015-08-05

**Authors:** María-Carmen Fernández-Pinos, Marta Casado, Gemma Caballero, Erik R. Zinser, Jordi Dachs, Benjamin Piña

**Affiliations:** 1 Department of Environmental Chemistry, IDAEA-CSIC, Barcelona, Catalonia, Spain; 2 Department of Microbiology, University of Tennessee, Knoxville, Tennessee, United States of America; Mount Allison University, CANADA

## Abstract

Newly designed primers targeting *rbc*L (CO_2_ fixation), *psb*A (photosystem II) and *rnp*B (reference) genes were used in qRT-PCR assays to assess the photosynthetic capability of natural communities of *Prochlorococcus*, the most abundant photosynthetic organism on Earth and a major contributor to primary production in oligotrophic oceans. After optimizing sample collection methodology, we analyzed a total of 62 stations from the Malaspina 2010 circumnavigation (including Atlantic, Pacific and Indian Oceans) at three different depths. Sequence and quantitative analyses of the corresponding amplicons showed the presence of high-light (HL) and low-light (LL) *Prochlorococcus* clades in essentially all 182 samples, with a largely uniform stratification of LL and HL sequences. *Synechococcus* cross-amplifications were detected by the taxon-specific melting temperatures of the amplicons. Laboratory exposure of *Prochlorococcus* MED4 (HL) and MIT9313 (LL) strains to organic pollutants (PAHs and organochlorine compounds) showed a decrease of *rbc*L transcript abundances, and of the *rbc*L to *psb*A ratios for both strains. We propose this technique as a convenient assay to evaluate effects of environmental stressors, including pollution, on the oceanic *Prochlorococcus* photosynthetic function.

## Introduction

Oceanic phytoplankton are responsible for almost a half of the global net primary production (NPP) [1], whereas marine picocyanobacteria account for 32 to 80% of primary production in the oligotrophic oceans [2–5]. *Prochlorococcus* is the smallest and most abundant photosynthetic organism known on Earth, ubiquitously found throughout the euphotic zone in tropical and subtropical oligotrophic oceans from 40° S to 40° N [6], with population abundances of about 10^5^ cells/mL [7–13]. Therefore, *Prochlorococcus* contributes significantly to the NPP in this latitudinal band [8, 14, 15], playing a relevant role in the global carbon cycle. There are two clades of *Prochlorococcus*, adapted to either high-light (HL) and low-light (LL) conditions [16]. They differ in a number of genetic and ecophysiological characteristics, including their divinyl-chlorophyll chl*b*_2_ / chl*a*_2_ ratios, ribosomal 16S rDNA sequences, and distributions in the water column [16–18]. Multiple genetic and physiologically different *Prochlorococcus* lineages have been so far characterized [17, 19, 20], showing distinct adaptations to solar irradiance, nutrients availability, and, consequently, different horizontal and vertical distributions [11, 13, 21, 22]. Preliminary data indicate that there are many *Prochlorococcus* lineages still to be characterized in the world oceans [23–29].

Although genetic variability of *Prochlorococcus* has been extensively studied in the ocean, these studies were mainly based on the distinct sequences of the 16S and 23S rDNAs and the 16S/23S rRNA internal transcribed spacer (ITS) [7, 20, 22, 28, 30, 31]. In contrast, analyses of functional gene expression are usually performed in laboratory conditions using pure cultures and specific probes for the target strains [32–37]. When functional genes have been studied in the field, the assessment has been restricted to a limited group of strains [38, 39].

In this paper, we focus on the photosynthetic activity of *Prochlorococcus* given its relevance as a primary producer in the global oceans. With this purpose, we designed new primers for two functional photosynthetic protein-coding genes, *rbc*L and *psb*A, and measured their mRNA abundances by quantitative PCR assays along a global circumnavigation sampling campaign. The gene *rbc*L encodes the large protein subunit of the RuBisCO enzyme, responsible of catalyzing the rate-limiting step of Calvin cycle. RbcL protein abundance has been considered a good representative for the activity of the entire Calvin cycle [40] and hence quantification of the *rbc*L mRNA levels is considered a useful proxy for the carbon fixation activity [38, 39, 41, 42]. While its transcription is mainly regulated by light intensity [41, 43, 44], it also correlates with other variables like nitrogen concentration [45]. The *psb*A gene encodes the photosystem II (PSII) core protein D1, which is the primary target of photo-inactivation and protect the cell from photo-oxidative stress [46]. The damage of D1 protein results in photo-inhibition, decrease of the photosystem II efficiency, and a drop of photosynthetic carbon fixation [47, 48]. Levels of the PsbA protein subunit have been shown to reflect the cellular PSII content [40]. We therefore consider the *psb*A transcript concentration as a proxy of the functionality of the PSII in the cell. Although *psb*A expression is mainly affected by light intensity [32, 44, 49] and UV radiation [48], it is also sensitive to variables such as iron starvation and glucose availability [50]. We selected the *rnp*B gene as endogenous standard of the quantification of the two target genes. This gene encodes the RNA component of RNaseP, a ubiquitous enzyme required for tRNA 5’ end maturation in prokaryotes. It has been previously reported as a suitable reference gene for *Prochlorococcus* in qRT-PCR analyses [44, 51–53], since its levels of mRNA remain stable at different conditions of irradiation, iron, phosphate, glucose, UV radiation, nitrogen, or light quality [32–37, 43, 52, 54, 55]. Aiming to integrate the high genetic variability of *Prochlorococcus* strains by a simple biomarker, we designed for each of the three tested genes, *rnp*B, *rbc*L and *psb*A, two separate sets of primers for HL and LL *Prochlorococcus*. A fundamental reason for the selection of these genes (and of the regions within them to be amplified) is their high sequence conservation among the different isolates of the genus, and their relative divergence from homologous sequences from other cyanobacteria. However, giving the close phylogenetic relationship between some LL *Prochlorococcus* and strains from the genus *Synechococcus*, at least some cross-amplification seemed *a priori* unavoidable [20, 28].

There are multiple environmental variables (light, nutrients, etc.) known to affect photosynthesis, and hence prone to alter the expression of both target genes [32, 41, 43, 44, 48, 49]. In this work, we selected persistent organic pollutants (POPs) as anthropogenic stressors of *Prochlorococcus* photosynthesis capacity, since they are broadly distributed over the globe and reach remote oceanic waters [56–58]. They accumulate in marine phytoplankton [59–64] decreasing cyanobacterial growth rate and biomass, inhibiting the PSII, and causing cellular bleaching and death for *Prochlorococcus* [65, 66].

The objectives of the present work were i) to develop a simple and high-throughput amenable methodology to quantify and detect changes in the expression of *rbc*L and *psb*A genes in *Prochlorococcus*; ii) to test the applicability and specificity of this methodology to both laboratory axenic cultures and field samples collected during an oceanographic cruise; and iii) to analyze the potential effects of POPs, under controlled conditions on *Prochlorococcus* pure cultures. The main goal is to generate a molecular tool applicable to natural communities to study the effects of the influence of diverse environmental stressors, and particularly anthropogenic ones, on the photosynthetic capacity of *Prochlorococcus* natural communities and, ultimately, on the oceanic carbon cycle.

## Material and Methods

### Optimization of sample collection and RNA extraction

We performed preliminary filtration tests using water samples from the North Western Mediterranean Sea (41 39.7 N 02 54.6 E). Based on previous estimation of *Prochlorococcus* mRNA half-live rates [67], we set a maximum operation time of 10 minutes from sampling to RNA stabilization, and all parameters were adjusted to this limit. We tested three different 47-mm-diameter, 0.2-μm-pore-size filters: Nucleopore polycarbonate (Whatman, Freiburg, GE), Durapore PVDF (Millipore, Billerica, MA) and Omnipore PTFE (Millipore). Finally, for each filter type we evaluated the saturation capacity, cell retention and ease of use for nucleic acids isolation.

### Design of primers and specificity checks using axenic cultures

Primers for *Prochlorococcus rbc*L, *psb*A and *rnp*B genes were designed *de novo* for both HL and LL clades (Table 1), starting from different known *Prochlorococcus* sequences ([68], listed in S1 Table) using Geneious 5.6.6, Biomatters (available from http://www.geneious.com/). We tested the specificity and efficiency of the designed primers by qRT-PCR analyses using total RNA from axenic cultures of *Prochlorococcus* MED4 and MIT9515 HL strains, MIT9313 and NATL2A LL strains, and *Synechococcus* WH7803 strain. Cultures were grown in artificial media for *Prochlorococcus* (AMP-J) [69], at 22°C in a natural diel light cycle incubator [69] with a maximum irradiance of 100 μmol quanta m^−2^ s^−1^. In addition, a culture of *Prochlorococcus* EQPAC1 strain [31] was obtained from the Roscoff Culture Collection and processed for nucleic acid analyses without further manipulation. All the strains were collected at approximately 4×10^5^ cells/mL, similar to the mean abundance of natural *Prochlorococcus* communities [8, 9, 15, 25]. For consistency, the *Synechococcus* WH7803 strain was collected at the same cell density, despite the fact that this genus is typically one or two orders of magnitude less abundant than *Prochlorococcus* [6, 70, 71]. The collection and storage procedure was as used for field samples, which is explained in detail below.

**Table 1:** Designed primers used for qRT-PCR analysis, and expected PCR fragment sizes.

Gene	Primer sequences (5’- 3’)		Amplicon size (bp)
*rnp*B-HL	Fp	GTGTTGGCTAGGTAAACCCCG	*81*
	Rp	ATCTACTTTTAAGCGCCGCTTG	
*rbc*L-HL	Fp	ATGGTCATCCATGGGGTTCAGC	*104*
	Rp	GGTCGCGAAATCGAAAAAGAGAGT	
*psb*A-HL	Fp	ACCAGTTTCAGCAGCTTTCGCA	*128*
	Rp	TGTTTTCCAGGCAGAGCACAACA	
*rnp*B-LL	Fp	TGCCACAGAAAMACACCGC	*106*
	Rp	GCATCGAGAGGTGCTGGC	
*rbc*L-LL	Fp	GAAGATATCCGCTTCCCGATGGC	*140*
	Rp	AAGCCAAAGCTTGGCCTTTCTGG	
*psb*A-LL	Fp	TCTGGTGCTGTTGTTCCTTCCAG	*200*
	Rp	GTATGCGCCCTTGGATCTGTGT	

Fp: Forward primer.

Rp: Reverse primer.

### Field sample collection and storage

**Fig 1:**
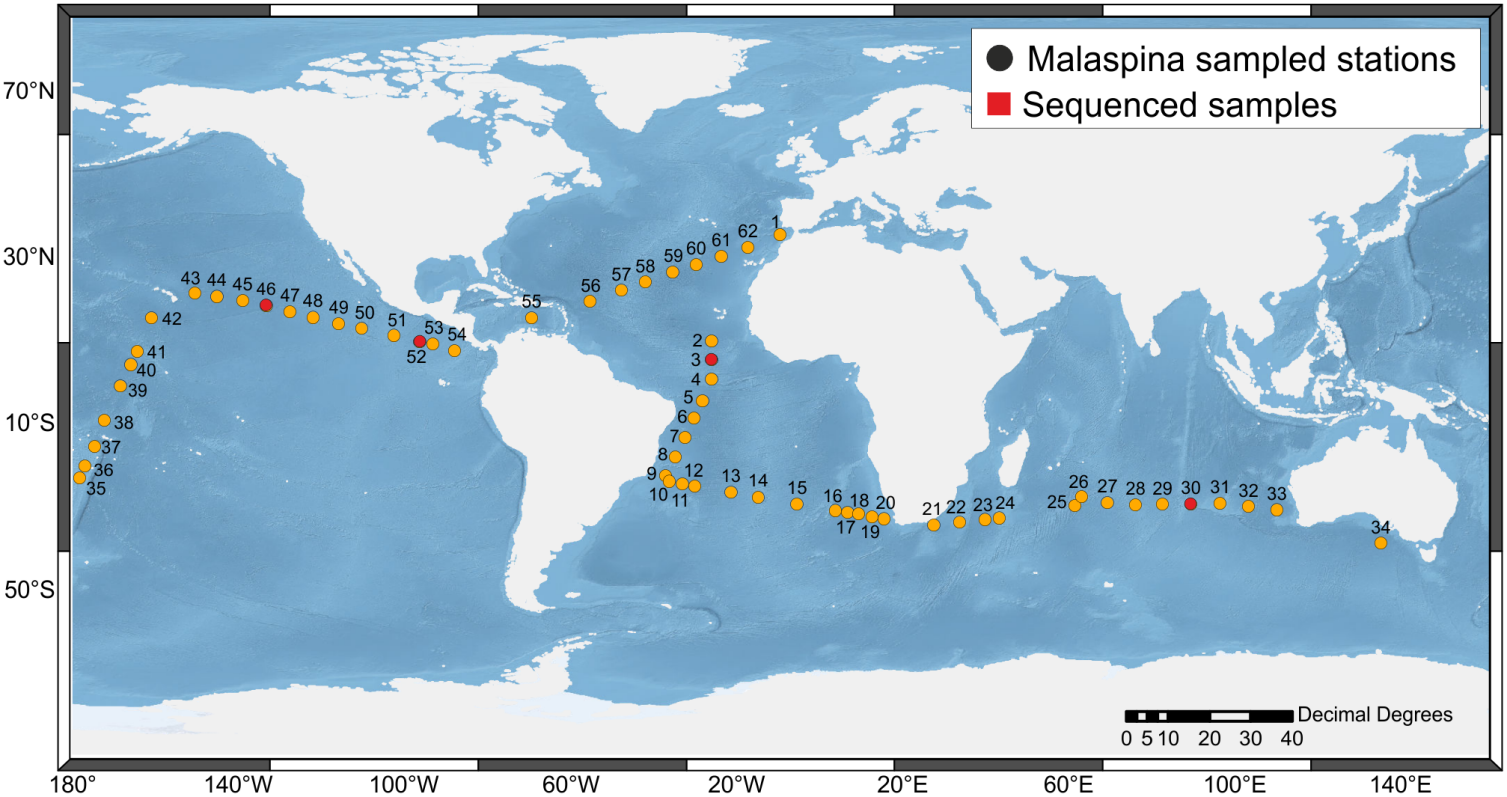
Oceanic stations from Malaspina 2010 circumnavigation selected for RNA analysis. Stations labeled in red correspond to those from which amplicon sequences were obtained.

A total of 182 samples of oceanic water were collected at 62 stations in the Atlantic, Indian and Pacific Oceans during the Malaspina 2010 circumnavigation, from 14 December 2010 to 14 July 2011 aboard the R/V *BioHesperides* (Fig 1 and S2 Table). We sampled three depths at each station: 3 m depth, (Niskin bottle), deep chlorophyll maximum (DCM) depth, and DCM+40 m depth (both in Niskin bottles attached to a rosette—CTD system). Samples were collected between 8 h and 12 h am local time (10 h 45 min ± 46 min, S2 Table). This time period coincides with the peak of carbon fixation by *Prochlorococcus*, which occurs between dawn and midday [43, 72], as well as with the maximal expression of *rbc*L and *psb*A genes [44]. One liter of seawater from each sample was transferred from the Niskin bottle to a glass bottle, previously rinsed with milli-Q water and a small volume of the sampled seawater, prefiltered onto a 20-μm-pore-size net, and finally filtered onto 47-mm-diameter, 0.2-μm-pore-size PTFE filter at 80 mbar vacuum pressure. Filters were split into two halves. One half was preserved in lysis buffer (50 mM Tris-HCl, 40 mM EDTA, 0.75 M Sucrose) and stored at -20°C for genomic DNA extraction. The second half was preserved in RNAlater (Sigma-Aldrich, Saint Louis, MO) for RNA isolation analysis and preserved at -80°C. In all cases, we kept rigorously the time limit of 10 min between sample collection and the storage of the filter.

### Experimental exposure of axenic cultures to pollutant mixtures

To test the sensitivity of the designed primers to the potential effects of environmental stressors of photosynthesis presumably inducing a small variation on the gene expression, we decided to perform experiments under controlled conditions using axenic cultures and commercial mixtures of organic pollutants. We selected the well-characterized MED4 and MIT9313 *Prochlorococcus* strains as representatives of HL and LL clades, respectively, since they differ in size, nutritional requirements, preferences in light intensity, and genome size [73]. We chose as stressors a mixture of the US Environmental Protection Agency 16 priority polycyclic aromatic hydrocarbons (PAHs) [74] (Dr. Ehrenstorfer, Augsburg, GE), and a mixture of five organochlorine pesticides (OClP), hexachlorobenzene (HCB), and hexachlorocyclohexane (HCH, α, β, δ, and γ isomers), given their toxicity, persistence, potential to bioaccumulate, and environmental relevance [57, 58, 75, 76]. Growing MED4 and MIT9313 cultures were challenged with either pollutant mixtures. The PAH mixture was added to a final concentration of 700 ng/L, the approximated concentration estimated to reduce growth of natural populations of *Prochlorococcus* by 10% (LC10) [66]. No equivalent toxicity data exist for the OClP mixture; thus, we used a final concentration of 500 ng/L that is known to have no effect on growth rate in the experimental conditions (not shown).

We performed 4 experiments, one for each combination of strain and pollutant mixture. For each experiment, we used a culture adapted previously to the experimental conditions, which was diluted to an approximate concentration of 10^5^ cells/mL. After two days, once it had reached a concentration of 2–4*10^5^ cells/ml, we split it into twelve 0.5 L flasks. 6 flasks where challenged with the pollutant mixture (treatments) and the other 6 where grown in regular conditions (controls). Three pairs of treatment and control flasks were collected after 30 minutes and the rest after 24 hours of exposure. Every experiment was started at 11 h local time, at the same point of the incubator diel cycle, when the radiation was still increasing before reaching the maximum, to avoid differences between experiments due to the effect of the diel cycle on the mRNA abundances of the target genes [44]. Cell concentration was measured at the beginning and at the end of the experiment with a flow cytometer (Guava easyCyte, Millipore), using the characteristic red auto-fluorescence of *Prochlorococcus* [77]. To analyze the content of chlorophyll *a*, a 50 mL aliquot of each culture was filtered through a GF/F filter (Whatman) under vacuum pressure and stored at -20°C until analysis. Samples for molecular analyses were collected and preserved as described above for field samples.

### DNA and RNA isolation

For DNA extraction, the corresponding half filters, once thawed, were incubated at 37°C for 45 minutes with a 5 mg/mL lysozyme solution in lysis buffer. Then, 0.5 mg/mL proteinase K and 100 μL of 10% sodium dodecyl sulphate were added and further incubated at 55°C for 1 h. DNA was extracted twice with phenol-chloroform-isoamyl alcohol (25:24:1; pH 8), and once with chloroform-isoamyl alcohol (24:1). Genomic DNA from the aqueous phase was then precipitated adding ammonium acetate to 0.4 M and one volume of isopropyl alcohol. After 20 minutes of incubation at -20°C, the precipitate was centrifuged and the supernatant was removed. The obtained DNA pellet was washed with 70% ethanol and finally suspended in 30 μL of TE (10 mM Tris, 1 mM EDTA; pH 8).

Total RNA was isolated from the RNA-stabilized filter using the mirVana kit (Ambion, Austin, TX) [78], after removing the excess of RNAlater. The final elution volume (about 100 μL) was concentrated by partial lyophilization to approximately 40 μL, and total RNA concentration was measured by spectrophotometric absorption at 260 nm with a NanoDrop ND-8000 spectrophotometer (NanoDrop Technologies, Delaware, DE). RNA quality was checked with an Agilent 2100 Bioanalyzer (Agilent Technologies, Santa Clara, CA). Total RNA was treated with DNase I (Ambion) to remove genomic DNA contamination and reverse transcribed to cDNA using First Strand cDNA Synthesis Kit (Roche, Mannheim, GE). Lastly, the resulting cDNA was stored at -20°C for further analyses.

### qRT-PCR analysis, cloning and sequencing of amplicons from cultures and field samples

Aliquots of genomic DNA (2.2 ng for culture samples or 5 ng for field samples), or total RNA (2.2 ng for culture samples or 3.75 ng for field samples), were used to quantify specific transcripts by qRT-PCR in a LightCycler 480 (Roche Diagnostics, Indianapolis, IN) thermocycler using SYBR Green Mix (Takara Bio Inc., Siga, Japan). After thermal activation at 95°C for 10 s, forty-five amplification cycles (95°C for 5 s, 60°C for 35 s), followed by a melting curve program (65–95°C with a heating rate of 0.11°C/s) and a final extension step at 60°C for 30 s. PCR efficiency values for the tested genes were calculated as described elsewhere [79]. When necessary, we redesigned the primers until reaching efficiencies between 95% and 105%. Relative mRNA or genomic DNA abundances of the different genes were calculated using the second derivate maximum of their respective amplification curves (Cp). All samples were run by duplicate, ensuring that the difference between the replicates was less than 0.25 cycles. Cp values are inversely correlated with the logarithm of the initial number of copies of the amplified DNA sequence, N_o_, following the equation

$${\text{N}}_{0}=\text{ k}{\left(1+\text{E}\right)}^{-\text{Cp}}$$

in which E is the efficiency of the reaction (equal to 1 if the primer efficiency is 100%), and *k* a coefficient related to the number of amplified molecules needed to detect the amplification product by the instrument. Therefore, high Cp values implicate low initial concentrations, and vice versa.

For relative gene expression analyses, we normalized the Cp values of the *rbc*L and *psb*A target genes (tg) to the corresponding values of the *rnp*B reference gene (ref) to obtain ΔCp values, ΔCp = Cp_ref_ − Cp_tg_, to account for differential cell concentrations and sample processing when quantifying gene expression. The ratios between treatments and controls mRNA/DNA levels were calculated from these ΔCp values, as

$$\frac{{\text{Copies}}_{\text{Treatment}}}{{\text{Copies}}_{\text{Control}}}=\;2^{{\Delta \text{Cp}}_{\text{Treatment}}-{\Delta \text{Cp}}_{\text{Control}}}$$

Similarly, the ratios between *rbc*L and *psb*A mRNA/DNA levels were calculated as

$$\frac{{\text{Copies}}_{rbc\text{L}}}{{\text{Copies}}_{psb\text{A}}}=\;2^{{\Delta \text{Cp}}_{rbc\text{L}}-{\Delta \text{Cp}}_{psb\text{A}}}$$

Melting temperature (Tm) values for each amplified product from each sample was calculated as the negative first derivation curve of the fluorescence intensity curve over temperature set in the PCR protocol (-dF/dT curve) [80]. Only amplifications with single peaks, denoting a single amplification product, were considered for quantification. PCR products of MED4 and MIT9313 cultures and field samples from 4 oceanic stations (Table 2) were cloned into the vector pTZ57R/T (InsTAclone PCR clone kit, Thermo Scientific, Waltham, MA) and propagated using XL-Blue competent cells. DNA sequencing was performed on 3730 DNA Analyzer (Applied Biosystems), and we compared the results to existing DNA sequences by the BLAST algorithm at NCBI server (http://www.ncbi.nlm.nih.gov/blast/Blast.cgi) [81]. We performed phylogenetic trees including the closest sequences identified in the BLAST analyses and the newly amplified sequences using Geneious 5.6.6, Biomatters (available from http://www.geneious.com) for sequence alignment and phylogenetic tree design. Final unrooted tree diagrams were drawn using the FigTree software (http://tree.bio.ed.ac.uk), and edited using the Corel Draw program (Corel Corporation, Ottawa, Ontario, Canada).

**Table 2:** Coordinates and collection data from four different oceanic stations sampled during Malaspina circumnavigation.

Sample Name	Station number	Location	Date (dd-mm-yy)	DCM depth (m)	Mixed layer depth (m)	Collection local time (h)		
						3m	DCM	DCM+40
**Atlantic**	3	05 0.40 N. 26 1.59 W	30-12-10	120	76	8:16	10:35	10:35
**Indian**	30	29 40.27 S. 89 26.46 E	05-03-11	135	36	8:05	10:12	10:15
**Pacific 1**	46	18 4.30 N. 133 19.27 W	22-05-11	125	94	7:16	10:32	10:34
**Pacific 2**	52	9 26.42 N. 96 20.17 W	04-06-11	19	18	7:20	10:09	10:08

### Chlorophyll analyses

Frozen filters were extracted in variable volumes (3–5 ml) of 95% acetone during 24 hours followed by sonication during 5 min at low temperature (4°C). Extracts were then centrifuged at 4000 rpm for 10 minutes to remove cell and filter debris. A 1.5-mL aliquot of the acetone extract was measured for chlorophyll using a UV-spectrophotometer. We assumed that the absorbance at 665 nm was linearly correlated to the total amount of divinyl-chlorophyll *a* [82].

### Statistical tests

Statistical tests were performed using the SPSS 19 (SPSS Inc., Chicago, IL) package, with additional calculations performed using the R package (http://CRAN.R-project.org/). Normality of data distributions was checked by the Kormogorov-Smirnov test. Differences on mRNA abundances at different sampling depths were tested by the ANOVA plus Tukey HSD post-hoc test. Effects on the *rbc*L and *psb*A gene expression or DNA abundance due to organic pollutant mixtures were tested by paired T-tests between treated and untreated cultures. A 3-way-ANOVA general linear model (GLM) was used to evaluate the differential effects of pollutants on cultures by comparison of the mean fold changes of the target genes of each treatment with respect to its control (mRNA Copies_Treatment_/Copies_Control_). Three categorical predictors were used in the GLM to assess the variability on gene expression: Treatment (PAH mixture *vs*. OClP mixture), Strain (MED4 *vs*. MIT9313) and Time of exposure (0.5 h *vs*. 24 h). Significance levels were set to 0.05.

## Results

### Methodological optimization

#### Sampling procedure

Comparison of filtration rates of PVDF, PTFE and PC filters under vacuum showed that only PVDF and PTFE filters maintained a filtration rate fast enough to filter 1 L of seawater in less than ten minutes, a time considered optimal to minimize changes on mRNA levels during sampling (graphs in S1 Fig). In addition, PTFE filters were compatible with both nucleic acid stabilization reagents used in this work (RNAlater for RNA and DNA lysis buffer for DNA), and did not interfere with the corresponding RNA and DNA extraction methods. A typical RNA extraction from 1 L of natural sea water yielded 100 to 3000 ng of DNA/RNA (average 900 ng of DNA/RNA), an amount considered sufficient for the intended analyses. Whereas PVDF and PTFE showed similar (and fast) filtration rates (S1 Fig), PTFE filters showed better RNA extraction efficiency and cell retention (S2 and S3 Figs).

#### Specificity of designed primers

qRT-analysis of cDNA samples from axenic cultures of HL *Prochlorococcus* (MED4, MIT9515 and EQPAC1) and LL strains (MIT9313 and NATL2A) showed a strong specificity of the designed primers (S3 Table). HL and LL strains amplified with the appropriate HL or LL primers showed Cp values at least 10 cycles lower than the values obtained with the non-appropriate LL or HL primers, which represents a 1000 fold difference in amplification efficiency. Amplicons from MED4 and MIT9313 amplified with HL and LL primer pairs, respectively, were sequenced and the results corresponded to the expected *rbc*L, *psb*A and *rnp*B sequences when analyzed by BLAST (Table 3). LL *rnp*B primers showed detectable amplification products when challenged with *Synechococcus* templates with similar efficiency than LL *Prochlorococcus* templates (S3 Table), whereas both LL *rbc*L and LL *psb*A primers gave Cp values more than 10 cycles higher for *Synechococcus* than for *Prochlorococcus*—again, a 1000-fold lower efficiency. The LL *rbc*L amplification products of *Prochlorococcus* and *Synechococcus* differed in their melting temperature (Tm), which ranged from 81°C to 86°C for LL *Prochlorococcus*, and from 88 to 89°C for *Synechococcus* strains (see S4 Fig and S3 Table), suggesting that this last amplicon corresponded to *Synechococcus*, although presenting a low-efficiency amplification. This characteristic can be used in field samples to detect the presence of *Synechococcus* or to evaluate sequence heterogeneity within a given sample. S4 Fig illustrates this possibility, as it shows the Tm profile from a cDNA preparation that combined cDNA of *Prochlorococcus* strain MIT9313 and *Synechococcus* strain WH7803 at equal cell concentrations.

**Table 3:** Characterization by BLAST of sequenced amplicons obtained using the designed primers on total RNA samples from *Prochlorococcus* MED4 and MIT9313 pure cultures.

Primers	Specie/ Strain	Query coverage	Identity	Accession no.
**MED4 (HL)**				
*rnp*B-HL	*Pro*. MED4	98%	100%	BX548174.1
	*Pro*. PCC9511	98%	100%	AJ272223.1
*rbc*L-HL	*Pro*. MED4	100%	100%	BX548174.1
	*Pro*. MIT9215	100%	93%	CP000825.1
*psb*A-HL	*Pro*. MED4	100%	100%	BX548174.1
	*Pro*. MIT9515	100%	99%	AY599030.1
	*Pro*. MIT9312	100%	99%	AY599028.1
	*Pro*. MIT9116	100%	99%	AY599031.1
	*Pro*. MIT9301	100%	98%	CP000576.1
	*Pro*. MIT9302	100%	98%	AY599029.1
**MIT9313 (LL)**				
*rnp*B-LL	*Pro*. MIT9313	100%	99%	BX548175.1
	*Pro*. MIT9303	100%	98%	CP000554.1
	*Syn*. WH8102	100%	93%	BX569689.1
	*Syn*. WH7803	100%	91%	CT971583.1
	*Syn*. CC9311.	100%	91%	CP000435.1
*rbc*L-LL	*Pro*. MIT9313	100%	100%	BX548175.1
	*Pro*. MIT9303	100%	96%	CP000554.1
	*Syn*. PCC7009	95%	81%	AM701777.1
	*Syn*. CB0209	95%	81%	AY452729.1
*psb*A-LL	*Pro*. MIT9313	100%	99%	BX548175.1
	*Pro*. MIT9303	94%	98%	AY599035.1
	*Syn*. CC9902	100%	88%	CP000097.1
	*Syn*. WH8102	97%	88%	BX569693.1

Pro. *Prochlorococcus*; Syn.: *Synechococcus*.

### Applicability of the developed methodology to *Prochlorococcus* natural communities

#### Analysis of mRNA abundance in oceanic populations of *Prochlorococcus*

Analysis of *rbc*L, *psb*A and *rnp*B Cp values in samples from the vertical profiles of 62 oceanic stations showed the expected distribution of HL and LL sequences at the three sampled depths (Table 4) [11, 18, 22]. In 3 m depth samples, amplification with HL primers occurred 5–10 cycles earlier than with LL primers. This indicates a concentration of the corresponding mRNAs 30 to 1000 times higher, according to the reverse correlation between Cp values and the mRNA concentrations. The reverse occurs at both DCM and DCM+40, in which LL primers showed lower Cp values than HL primers (Table 4). If we consider *rnp*B level as a direct indicator of the concentration of metabolically active cells, the data suggest that HL strains represent some 97% of the total *Prochlorococcus* cells at 3 m depth, a mere 6% at DCM and from 1 to 2% at DCM+40 (Table 4). A characteristic of these data is their relatively low variation coefficient, less than 10% in most cases. If we consider that they correspond to samples from three oceans and collected in a period of seven months, this represents a remarkable homogeneity of *Prochlorococcus* populations across the globe.

**Table 4:** Descriptive statistics of Cp values corresponding to the different primer pairs, stations, and depths sampled during Malaspina Circumnavigation.

	3 m		DCM		DCM+40	
	mean ± sd	n	mean ± sd	n	mean ± sd	n
*rnp*B-HL	20.98±2.34	61	22.65±2.73	61	26.17±2.57	60
*rbc*L-HL	21.09±2.35	61	24.05±2.8	61	28.71±2.33	60
*psb*A-HL	16.87±2.38	61	19.85±2.71	61	23.64±2.31	60
*rnp*B-LL	26±3.09	61	18.8±1.58	61	20.67±2.25	60
*rbc*L-LL	31.83±3.14	61	23.22±2.63	61	24.84±2.72	60
*psb*A-LL	25.07±3.85	61	17.38±2.51	61	20.02±2.85	60

**Fig 2:**
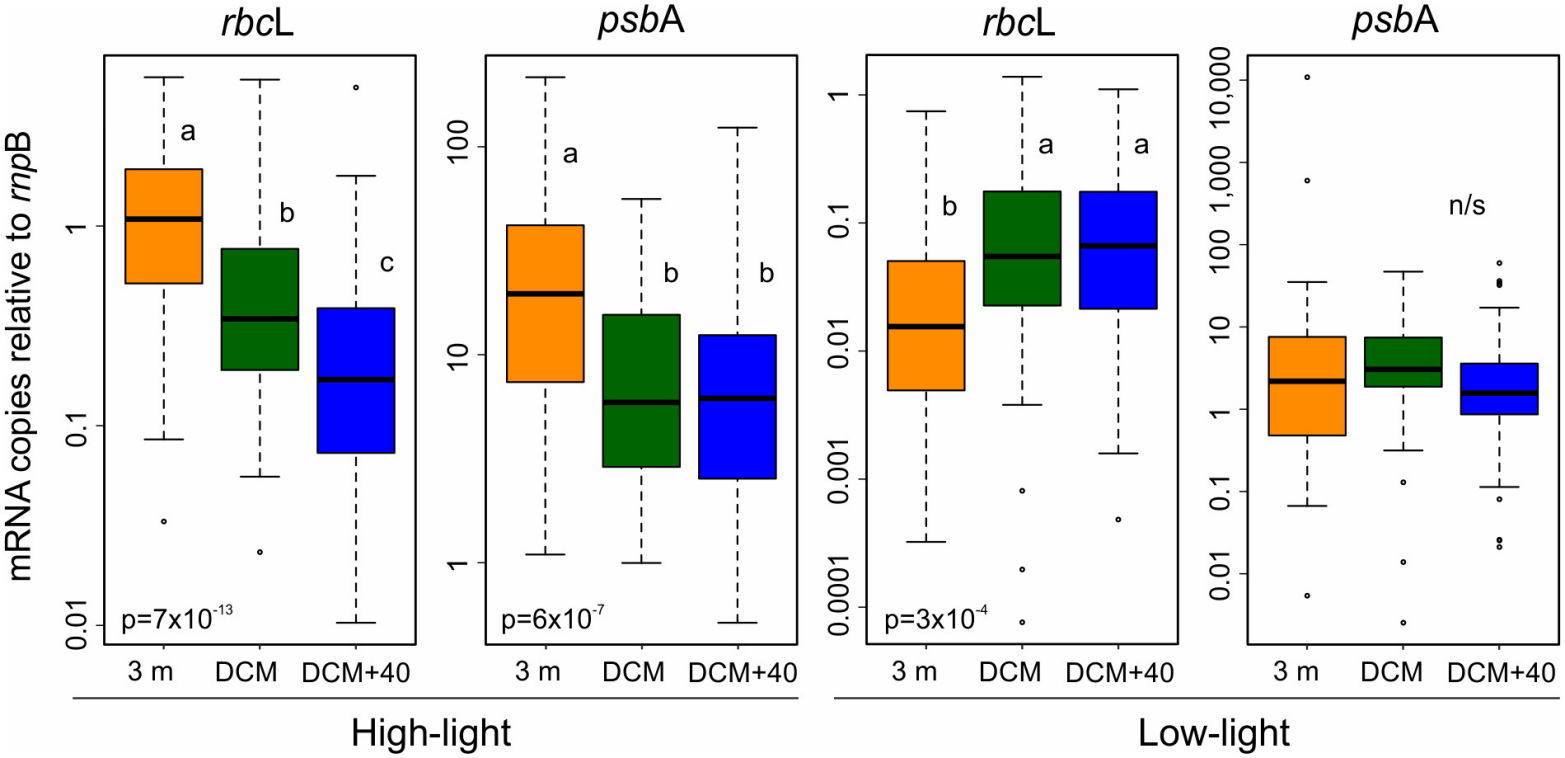
Relative expression of *rbc*L and *psb*A in surface (orange), DCM (green) and DCM+40 m (blue) samples for HL (left) and LL (right) clades. The boxes indicate data distribution parameters: range (whiskers), 25 to 75 percentiles (boxes) and median (thick horizontal bar). Circles represent outliers. Letters indicate statistically different distribution (ANOVA plus SD Tukey post hoc test); the corresponding p values are indicated at the bottom.

Fig 2 shows the global distributions of relative abundances of *rbc*L and *psb*A, normalized by the reference gene *rnp*B, for the two clades and at the three sampled depths. The relative mRNA abundances of both HL genes decreased with the depth, being maximal at 3 m depth and minimal at DCM+40. On average, 3 m depth samples showed five times more HL *rbc*L, and three times more HL *psb*A mRNA copies than DCM+40 samples (Fig 2). LL genes showed the opposite tendency, although the effect was less clear. LL *rbc*L mRNA was about three times more abundant at DCM, or DCM+40, than at 3 m depth samples, whereas no significant differences were observed for LL *psb*A, in part due to the high dispersion of the values at 3 m depth (Table 4).

#### Analysis of mRNA sequence heterogeneity in oceanic samples

**Fig 3:**
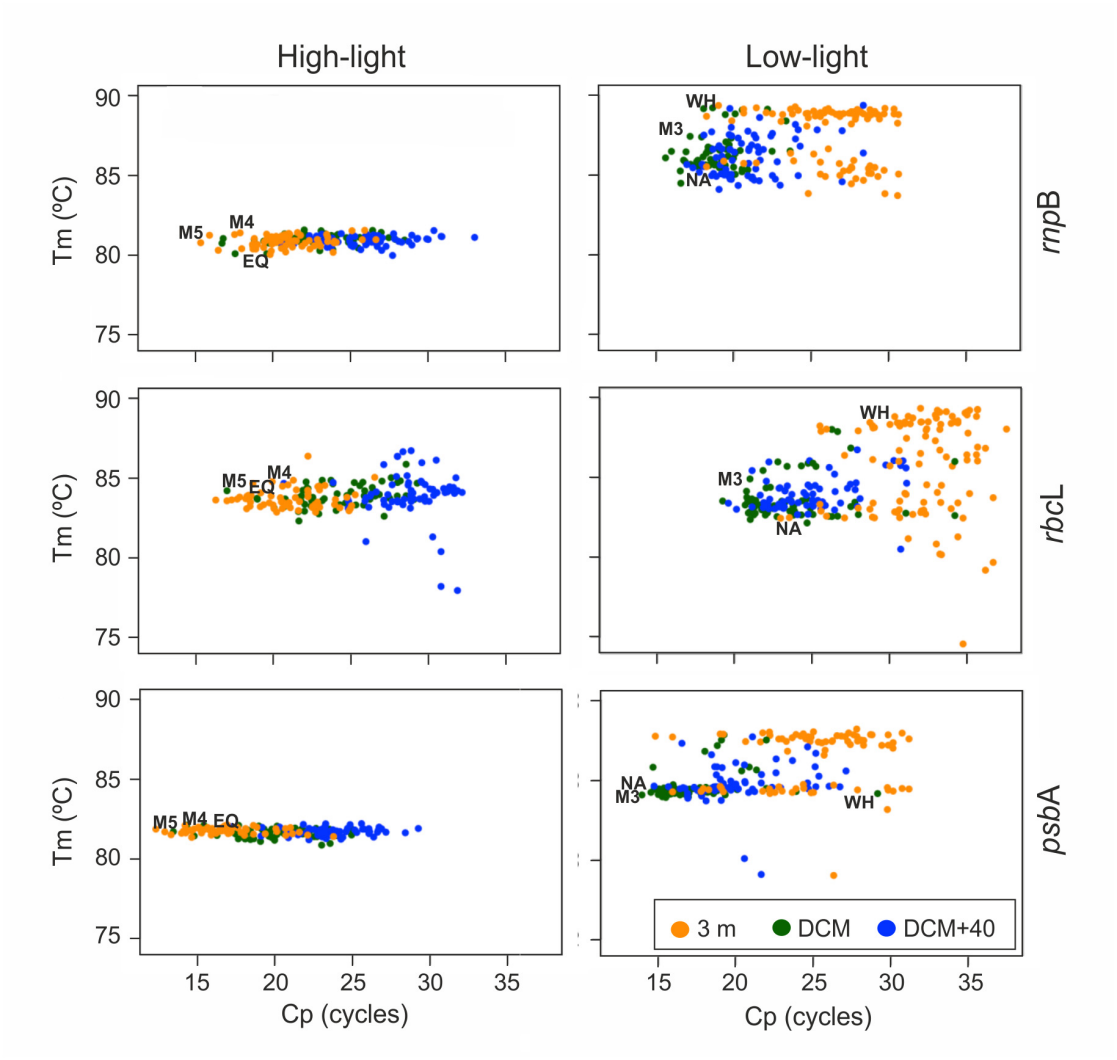
Representation of amplicon melting temperature (Tm) values, in°C, plotted against Cp values (in cycles) obtained for field samples at the three sampled depths: Surface (orange), DCM (green) and DCM+40 (blue). For comparison, the graphs include the corresponding values from supplementary S3 Table. HL amplicons: *Prochlorococcus* strains MED4 (M4), MIT9515 (M5) and EQPAC1-C (EQ). LL amplicons: *Prochlorococcus* strains MIT9313 (M3), NATL2A (NA), and the *Synechococcus* strain WH7803 (WH).

A combined analysis of Tm values of the different amplicons and their corresponding Cp values was used to estimate the sequence heterogeneity (and hence, genetic variability) within each sample (Fig 3). *rnp*B and *psb*A HL amplicons showed an extreme homogeneity of Tm values for all 182 samples, irrespective of the depth or of their relative abundance (Fig 3). In fact, all samples showed essentially the same Tm value, 81 and 82°C, respectively, within a margin of few tenths of°C (Table 5). The situation was similar for HL *rbc*L amplicons, although some samples, particularly at DCM+40, showed clearly differentiated Tm values (Fig 3, central left panel). In this case, 13% of DCM+40 samples showed enough HL *rbc*L amplicon heterogeneity to be resolved in two Tm peaks, a phenomenon that was only episodic in samples from 3 m or DCM and unobserved with the other two HL amplicons at any depth (Table 5). These atypical amplicons corresponded to samples with very low abundance in HL sequences—i.e., high Cp values (Fig 3). However, we consider that this Tm variability is still consistent with the natural variation of *Prochlorococcus* strains, as HL *rbc*L amplicons with a low Tm value were also observed for the cultured EQPAC1-C strain (marked as "EQ" in Fig 3, S3 Table in supplementary material).

**Table 5:** Descriptive statistics of Tm values of amplicons from field samples.

	Cultured strains		Field samples									
	Tm values (°C, x±sd)^a^				%Double peaks				% Non-average major peaks^b^			
Primers	*Prochlorococcus*	*Synechococcus*	Tm values (°C, x±sd)	*n*	All	3 m	DCM	DCM+40	All	3 m	DCM	DCM+40
*rnp*B-HL	81.0 ± 0.3	81.3	80.9 ± 0.3	182	0.0	0.0	0.0	0.0	3.3	4.9	3.3	1.7
*rbc*L-HL	83.4 ± 2.4	84.4	83.8 ± 0.7	182	5.5	1.6	1.6	13.3	2.7	0.0	1.6	6.7
*psb*A-HL	81.7 ± 0.1	81.4	81.7 ± 0.2	182	0.0	0.0	0.0	0.0	3.8	0.0	8.2	3.3
*rnp*B-LL	87.5 ± 0.7	88.1	86.7 ± 1.5	182	0.5	1.6	0.0	0.0	0.0	0.0	0.0	0.0
*rbc*L-LL	83.2 ± 2.5	89.7	84.4 ± 2.0	182	8.8	26.2	0.0	0.0	7.7	23.0	0.0	0.0
*psb*A-LL	85.6 ± 2.3	82.4	85.3 ± 1.5	182	1.1	0.0	0.0	3.3	1.1	3.3	0.0	0.0

^a)^ Separated values for each clade. Values from S2 Table.

^b)^ Percentage of major peaks statistically different (2*sd) from the corresponding average Tm values.

LL amplicons showed much higher sequence heterogeneity than their HL counterparts (Table 5, Fig 3, right panel). The corresponding graphs in Fig 3 show up to three distinct amplicon populations, particularly at 3 m depth. Also in this case, the highest variability corresponded to samples with low amplicon abundance (i.e., higher Cp values). We attributed a substantial fraction of this amplicon variability, at least at 3 m depth, to the presence of *Synechococcus*, as illustrated by the distribution of the LL *rbc*L amplicon in the central right panel of Fig 3. This particular panel shows a cluster of surface samples at high Cp values and high Tm values, similar to the ones observed for the cultured *Synechococcus* strain WH7803 (marked as WH in Fig 3, S3 Table). However, the data also suggest the presence of some LL *Prochlorococcus* cells. Up to 26% of surface samples showed double Tm peaks for the LL *rbc*L amplicon, one of the peaks coinciding with the typical Tm values for *Prochlorococcus* (Table 5). The other two LL genes do not allow distinction between the two genera, LL *Prochlorococcus* MIT9313 strain (M3 in Fig 3) shows amplicons with essentially the same Tm as their *Synechococcus* counterparts (Fig 3, S3 Table).

**Fig 4:**
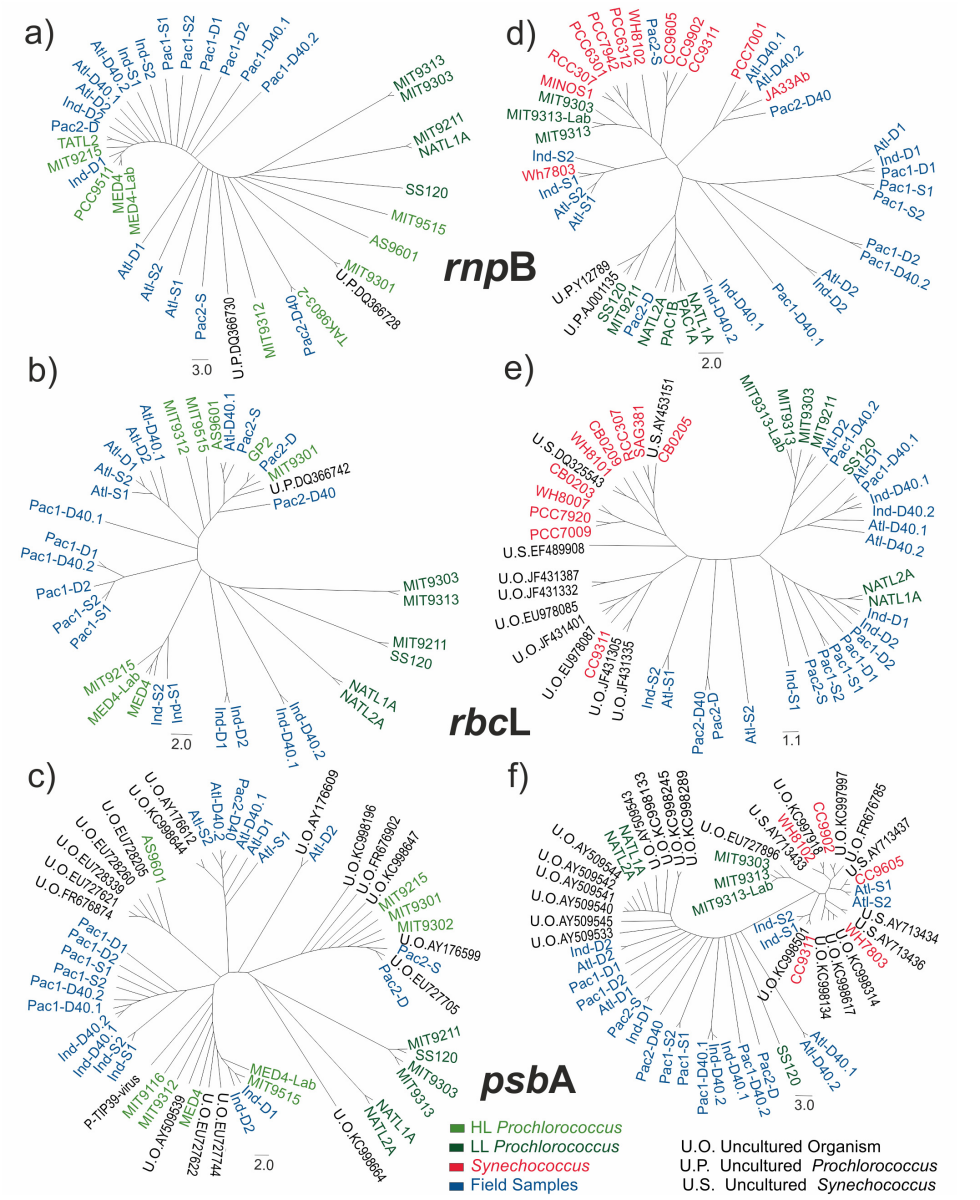
Cladograms of sequences from amplicons derived from field and laboratory samples compared to the closest sequences identified by BLAST. Two qRT-PCR products were sequenced for each sample from Atlantic, Indian and Pacific 1 stations, and only one for Pacific 2 samples (more information in supplementary S4 Table). Amplicons from surface, DCM, and DCM+40 samples from the different stations are identified with "S", "D" and "D40" letters. Panels a-c correspond to HL amplicons, whereas panels d-f correspond to LL amplicons. For each ecotype, cladograms corresponding to *rnp*B (a,d), *rbc*L (b,e) and *psb*A (c,f) genes are presented. The trees also include sequences from the GenBank. LL *Prochlorococcus*, HL *Prochlorococcus*, and *Synechococcus* (Syn.) sequences are indicated by dark green, light green and red colors, respectively. Amplicons from MIT9313 and MED4 strains grown in the lab were also sequenced and included in the analysis as MIT9313-Lab and MED4-Lab, respectively. Sequences for uncultured microorganism isolates are marked as U.P. (identified as *Prochlorococcus*), U.Syn. (identified as *Synechococcus*) or U.O, (not identified).

Sequence analysis of amplicons from four different stations at the three sampled depths demonstrated that they all encoded the targeted genes (Fig 4 and supplementary S4 Table). Sequences from HL amplicons were very similar in all cases to reported sequences from HL *Prochlorococcus* strains belonging to the eMED4 and eMIT9312 ecotypes (S4 Table). Similarly, amplicons from LL primers showed strong sequence similarities to LL *Prochlorococcus* strains included into the eMIT9313, eMIT9211, eNATL2A, and eSS120 ecotypes [20]. However, ten out of the 21 sequenced LL amplicons from surface samples were identified as *Synechococcus* sequences (S4 Table). This is keeping with the predominance of *Synechococcus* strains in conditions of high solar irradiance [10–12, 16–18, 22], and corroborates our interpretation of the amplicon Tm variability observed in Table 5 and Fig 3.

The cladograms in Fig 4 compare the 126 sequenced amplicons with their closest sequences present in GenBank. For HL *rbc*L and *psb*A amplicons (left panels), field samples tend to form small clusters, loosely related to their geographical origin (Fig 4b and 4c). The relative position of these clusters and bona fide HL *Prochlorococcus* sequences suggests that the variability observed among field samples is similar to the one observed in natural HL *Prochlorococcus* isolates. For example, Fig 4b and 4c show most field sequences placed between MIT9301 and MED4. The same conclusion applies to HL *rnp*B sequences, despite the different topology of the corresponding cladogram (Fig 4a). Notice that sequences from LL *Prochlorococcus* strains (MIT9303, MIT9313, MIT9211, SS120, NATL1A and NATL2A) have been added to these HL cladograms as outgroups. They appear in separate branches in all three cladograms.

Cladograms for LL amplicons showed a clear distinction between *Synechococcus* and LL *Prochlorococcus* sequences for *rbc*L and *psb*A (Fig 4e and 4f). Consistently with the BLAST analyses, most field samples clustered with known *Prochlorococcus* sequences, with the exception of some samples from 3 m depth, which appear placed in *Synechococcus* branches. The topology of the LL *rnp*B cladogram differed from the other two, as it did not show a clear distinction between *Synechococcus* and *Prochlorococcus* sequences (Fig 4d). As a general conclusion, these cladograms indicate that all DCM and DCM+40 LL sequences corresponded to LL *Prochlorococcus* strains, whereas the results from 3 m samples showed a mixture of *Synechococcus* and LL *Prochlorococcus* sequences.

### Effects of pollutant mixtures on *Prochlorococcus* gene expression

**Fig 5:**
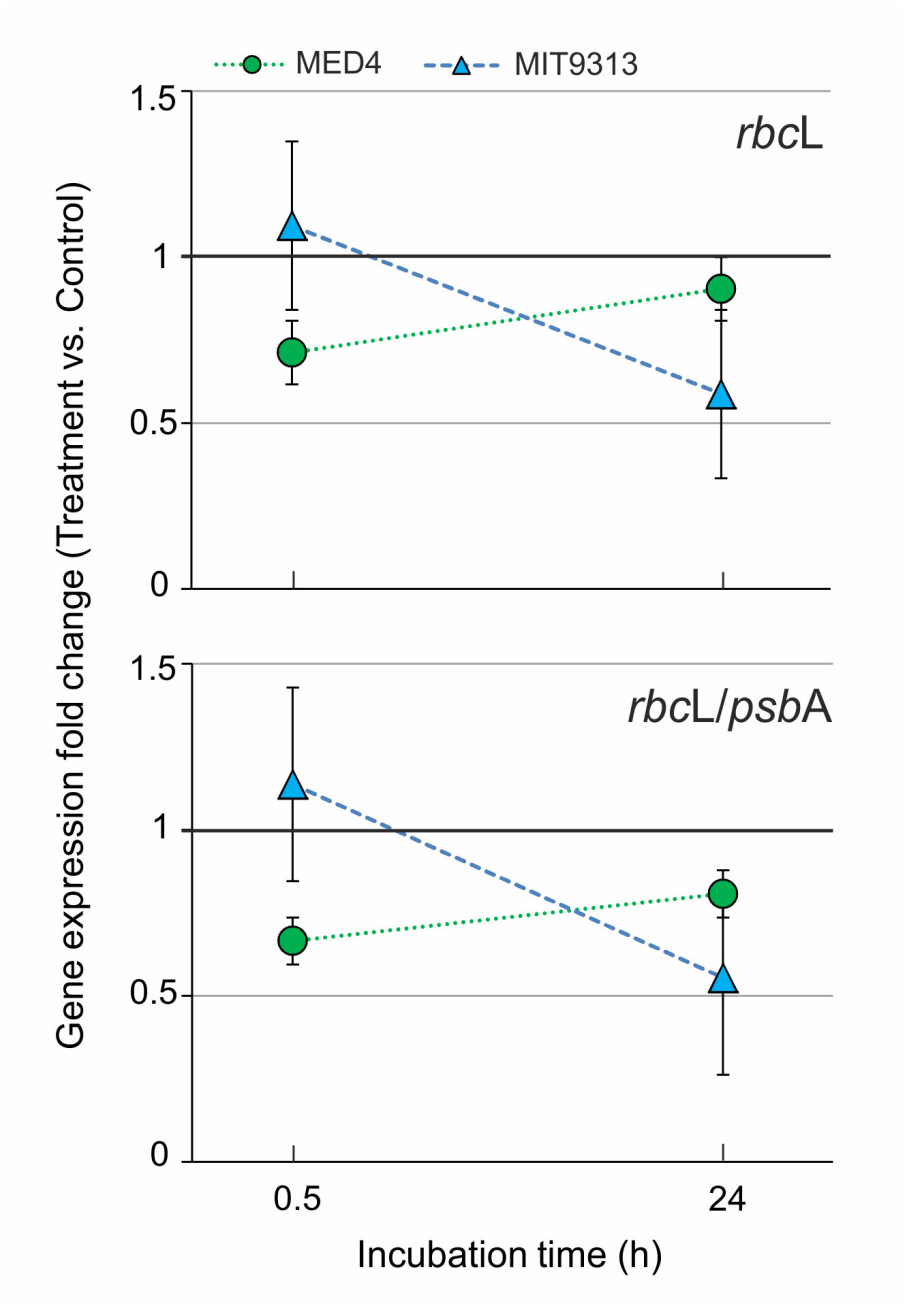
Graphic representation of the different temporal responses of MED4 (green circles) and MIT9313 (blue triangles) to pollutants. Results from PAH and OClP treatments are pooled together. Significant differences in mRNA abundance between treated and untreated cultures are marked with asterisks (*, p<0.05; **, p<0.01). Whiskers indicate 95% confidence limits.

Exposure of experimental *Prochlorococcus* MED4 and MIT9313 cultures to either PAHs or OClP mixtures did not affect significantly growth rates or chlorophyll contents (S5 and S6 Tables). However, treated cultures showed a decrease of the *rbc*L mRNA levels relative to control cultures, an effect not observed for *psb*A (Table 6). Combining data for all treatments, *rbc*L mRNA levels were reduced by 20% relative to the reference gene, a moderate, but significant decrease (Table 6). These data suggest that pollutants may alter the relative expression of photosynthetic genes, an effect likely to be added to the influence of other natural effectors we observed in the natural oceanic populations sampled at different depths (Fig 2). The analysis of the influence of different parameters on the effects of pollutants in *rbc*L expression (3-way ANOVA) indicated “time of exposure” as the only single factor with significant between-subject effects, as well as an interaction between “time of exposure” and “strain” (Table 6). These interactions can be visualized in Fig 5, which shows a decrease of the *rbc*L expression and of the *rbc*L/*psb*A ratio only after 24 hours of treatment for MIT9313, whereas for MED4 both values decreased during the first 0.5 h of exposure, recovering afterwards. While these results should be regarded only as indicative, they suggest that different *Prochlorococcus* strains may have different susceptibilities to organic pollutants, and that their temporal response to them may also differ.

**Table 6:** Mean results from paired t-tests between treatment and their respective controls (ΔCp_Treatment_ vs. ΔCp_Control_) and from the General Linear Model (GLM) performed with the fold changes (Copies_Treatment_ /Copies_Control)_, setting as fixed factors of GLM “Treatment” (PAH, OClP), “Strain” (MED4, MIT9313) and “Time” (0.5 h, 24 h) for each of the target genes and the ratio between them.

			*rbc*L	*psb*A	*rbc*L/*psb*A
	Value/Factors	df	Mean ± SD^a^		
**Paired T-test**	Ratio (T/C)^b^	21	0.82 ± 0.41 **	1.09 ± 0.45	0.78 ± 0.29 ***
			F^a^		
**GLM**	Treatment	1	0.292	0.082	2.165
	Strain	1	0.083	0.410	0.338
	Time	1	0.727	0.137	4.762 *
	Treatment * Strain	1	0.130	0.110	0.006
	Treatment * Time	1	0.354	1.983	1.828
	Strain * Time	1	3.349	0.097	14.840 **
	Treatment * Strain * Time	1	0.163	0.039	1.153

^a)^ *, p<0.05;

**, p<0.01;

***, p<0.001.

^b)^ Treatment versus control.

None of these differences between treatments and controls were observed when DNA, rather than RNA, copies were evaluated (supplementary S7 Table, compare to Table 6), which confirms that the observed effects on mRNA levels were indeed reflecting changes in the expression of the target genes and not derived from gene alterations or cell abundances. We concluded that *Prochlorococcus* can be affected by relatively low concentrations of pollutants, and that *rbc*L expression and the *rbc*L/*psb*A ratio may be sensitive indicators of these effects even in conditions in which the overall growth and chlorophyll concentration remain unaffected.

## Discussion

The ubiquitous presence of *Prochlorococcus* in tropical and subtropical oceans makes it extremely important for oceanic and global change studies. However, any global study on this genus needs to overcome the striking genetic variability of *Prochlorococcus* strains. This is especially important when using DNA or RNA-based markers, for they require exact base sequences for adequate amplifications. Our strategy in designing qRT-PCR primers was based in three considerations. First, the targeted genes had to be representative of the main physiological/ecological functions of *Prochlorococcus*. We selected photosystem functioning and carbon fixation, since these functions are intimately related to the major ecological function of *Prochlorococcus* as a main global primary producer, and because the photosynthetic machinery is at the core of the distinction between *Prochlorococcus* and other cyanobacteria. Secondly, we chose *psb*A and *rbc*L as representatives for PSII functionality and RuBisCo activity, respectively, as these two genes have been particularly well studied and sequenced for many cultured and non-cultured *Prochlorococcus* strains. Finally, we selected sequence regions that were particularly well conserved among *Prochlorococcus* strains and different from homologous regions in other cyanobacteria. However, the complexity of *Prochlorococcus* populations made it necessary to design two complete sets of primers targeting HL and LL ecotypes. Thus, we designed two sets of primers and tested them in real oceanic samples to analyse relative abundances and sequence heterogeneity. We tested the sensitivity of the developed markers in detecting physiological alterations induced by environmental pollutants in controlled experiments. Our ultimate goal was to generate a molecular tool applicable to natural communities to study the effects of global change vectors on the oceanic carbon cycle.

Unlike DNA, which is relatively stable and that can be found in cellular debris and other dead materials, RNA is easily degraded and only found in living cells. In addition, RNA synthesis (transcription) is a highly controlled process and usually reflects the environmental inputs on the cell metabolism. Therefore, analysis of RNA levels gives information not only of the presence of the cells, but also of their physiological status and metabolic activity. On the other hand, the very rapid turnover of mRNA in prokaryotes makes it difficult to obtain mRNA samples truly representative of the original (i.e., undisturbed) physiological state. To circumvent this problem, we limited manipulation times to an operational minimum and maximized the integrity of the collected RNA by the use of PTFE filters, relatively small volumes of water (1 L), and preserving filtered samples in RNAlater. This strategy allowed to obtain RNA preparations concentrated enough for qRT-PCR analyses and with no signs of RNA degradation.

As for most photosynthesis genes in *Prochlorococcus*, abundances of mRNA for *psbA* and *rbcL* exhibit significant periodicity over the light:dark photoperiod [44, 49]. *rbcL* mRNA peaks at sunrise, while *psbA* peaks at noon. Given these strong and not identical diel periodicities, we were careful to collect field and culture samples at the same time of day (morning). Consequently, *psbA*:*rnpB*, *rbcL*:*rnpB*, and/or *psbA*:*rbcL* ratio data from samples collected at other times of the day may thus not be directly comparable to our data, and will need to be interpreted with caution.

A major issue when analyzing gene expression in *Prochlorococcus* in field communities is to differentiate their mRNA sequences from those from other cyanobacteria and, at the same time, to deal with the large genetic variability of the genus. The designed method allows quantification of mRNA levels of the selected genes belonging from a variety of *Prochlorococcus* strains, because the designed primers are general enough to amplify sequences of close related strains within the genus. As in previous works [28], *Synechococcus* cross-amplifications may occasionally occur, but it can be detected by using the differential Tm values of LL *rbc*L amplicons from both species. This improvement allows a fast and inexpensive method for the detection of cross-amplification that solves a major issue of the genetic similarity between these two genera, and simplifies the markers to just 6 pairs of primers instead of specific primers and/or probes for each strain [32–34, 37]. This simplification is of great importance when using large sets of samples, as in our survey of samples from the Malaspina expedition.

More than 90% of the 126 amplicons sequenced from oceanic samples corresponded to *Prochlorococcus* strains, as judged by sequence homology. The only ten cases in which the amplified fragment encompassed *Synechococcus* sequences corresponded to 3 m depth samples amplified with LL primers, a combination that in almost all cases result in very high Cp values—that is, low specific mRNA levels. The analysis of Tm profiles of the amplified fragments showed that most samples presented single peaks with Tm values close to the expected *Prochlorococcus* values. Again, most exceptions belonged to surface samples amplified with LL primers, in which the appearance of Tm values corresponding to *Synechococcus* amplicons was very usual. Combining both types of results, we concluded that the designed primers amplified the expected *Prochlorococcus* sequences, with very little cross amplification, except for *Synechococcus* strains, found only in surface samples and only with LL primers.

Our results indicate variations in the expression of photosynthetic genes in response to external effectors. In natural HL populations, both *rbc*L and *psb*A showed their maximal relative mRNA levels in 3 m samples, where the conditions of light and oligothrophy were optimal, and minimal levels when they are most unfavorable—at DCM+40. The effect is not so clear for the LL clade, although the *rbc*L gene mRNA was significantly underrepresented in 3 m samples, an unfavorable environment for these strains. It is also noticeable the relationship between poor environmental conditions and sequence heterogeneity. In both clades, the minimal variability (at least in terms of Tm values, arguably a not particularly sensitive parameter) corresponded to the optimal growth conditions (3 m for HL, DCM for LL strains). If we interpret the reduction in photosynthetic gene expression as acclimatization to suboptimal light and/or temperature conditions, strains variability can similarly be interpreted as an adaptation phenomenon, in which different ecotypes may occupy specific niches in the water column.

On top of the natural variation in the relative gene expression of photosynthetic genes, the results from experimental exposures of *Prochlorococcus* to pollutant mixtures suggest that *rbc*L mRNA levels tend to decrease upon the presence of pollutants. This effect was observed in both the LL and HL cultured strains, which showed some differences on the timing and amplitude of the response. Our data also indicates that the ratio between *rbc*L and *psb*A mRNA levels is particularly sensitive to the presence of pollutants. A recent paper demonstrates that the ratio between RuBisCo (RbcL) and PSII (PsbA) molar ratio is strongly correlated to the capacity for electron transport away from PSII, suggesting a limitation of the electron transport rate by the RuBisCo to PSII ratio [40]. This fact may determine the use of photosynthesis rate for carbon fixation or for ATP generation. While it is not possible to directly translate messenger levels to protein concentrations or activities, it is conceivable that the presence of pollutants may increase energy consumption for the cell (for example, for detoxification or extra metabolic activity), reducing its capacity to fix CO_2_ and, therefore, the requirement for RuBisCo activity. Further experimental data is needed to test this hypothesis; however, we propose that both *rbc*L expression and the *rbc*L/*psb*A ratio may be useful indexes of the physiological status of the populations, likely responding to different environmental stressors.

We conclude that the proposed methodology allows not only quantifying the clade-specific photosynthetic potential of *Prochlorococcus* communities in the oceans, but also the assessment of changes in the gene expression due to environmental stressors, as light and/or nutrient conditions, and pollutants. The protocols for sample harvesting and preservation are adequate to ensure the integrity of RNA samples, and the qRT-PCR methodology allows processing of large number of samples at a reasonable cost. The developed methodology for quantifying the gene expression of the *rbc*L and *psb*A genes of *Prochlorococcus*, therefore, represents a first step for a future field and laboratory assessment of the different drivers and stressors affecting the photosynthetic function in *Prochlorococcus*, an essential issue for our understanding of the marine carbon cycle and its modulation in the current scenario of global change.

## Supporting Information

S1 Fig Permeability tests. Three filters of 0.2 μm pore-size and 47 mm of diameter made of different materials were used. Aliquots of 1 L of a seawater sample collected on 08/11/10 in Mediterranean Sea (41 39.7 N 02 54.6) were filtered onto each filter.
(TIF)

S2 Fig DNA recovery. Two water samples of Mediterranean Sea (41 39.7 N 02 54.6) were collected on 11/11/2010 (Nov) and 16/12/2010 (Dec), respectively, using three filters of 0.2 μm pore size and 47 mm of diameter made of different materials. 1 L of water was filtered through each filter.
(TIF)

S3 Fig RNA recovery. RNA isolated from both the filter and the pellet resulting from centrifugation of RNAlater where the filter was immersed. Three replicates of 1 L of Mediterranean Sea (41 39.7 N 02 54.6) water filtered onto Ominipore (PTFE) filters are shown. RNA concentration obtained from Omnipore (PTFE) filter is between two and three fold that obtained from the pellet.
(TIF)

S4 Fig Genetic marker for *Synechococcus*. The graphs shows melting temperature curves of amplicons using *rbc*L LL primers. Melting peaks for *Prochlorococcus* str. MIT9313 and *Synechococcus* str. WH7803 in the same sample are shown
(TIF)

S1 Table Model strain list. *Prochlorococcus* and *Synechococcus* strains used to design specific primers for *Prochlorococcus* of the selected genes by sequences alignments using Software *Geneious*.
(DOCX)

S2 Table Coordinates and collection data of field samples collected during Malaspina cruise. (DOCX)

S3 Table Primer specificity. Specificity of the designed primers checked by qRT-PCR using pure cultures of some *Prochlorococcus* and *Synechococcus* strains at equal cell concentrations and verification of the method validity in natural communities*.
(DOCX)

S4 Table Amplicon sequences. Sequencing of single amplicons from samples collected at three different depths and three sampled station during Malaspina circumnavigation.
(DOCX)

S5 Table Cell concentration variability. Paired-sample Wilcoxon signed rank tests achieved with control- treatment pairs of cell concentration measured.
(DOCX)

S6 Table Changes in chlorophyll a concentrations. Results from comparing means test achieved with control- treatment pairs of chlorophyll a fluorescence.
(DOCX)

S7 Table Quantitative Genomic changes. Relative changes in DNA abundance for different genes in MIT9313 and MED4 *Prochlorococcus* cultures when challenged with organic pollutant treatments (PAHs or OClP), evaluated at two different incubation times (0.5 and 24 hours). Results from four different paired T-tests are shown: one of the whole data set (“All samples”) and one of each data subset (“Strain”, “Treatment” and “Incubation time”).
(DOCX)
