# Supplementary figures and images for: Clade-Specific Quantitative Analysis of Photosynthetic Gene Expression in Prochlorococcus
Source: PLoS One. 2015 Aug 5;10(8):e0133207. doi: 10.1371/journal.pone.0133207 (PMC4526520; doi:10.1371/journal.pone.0133207)

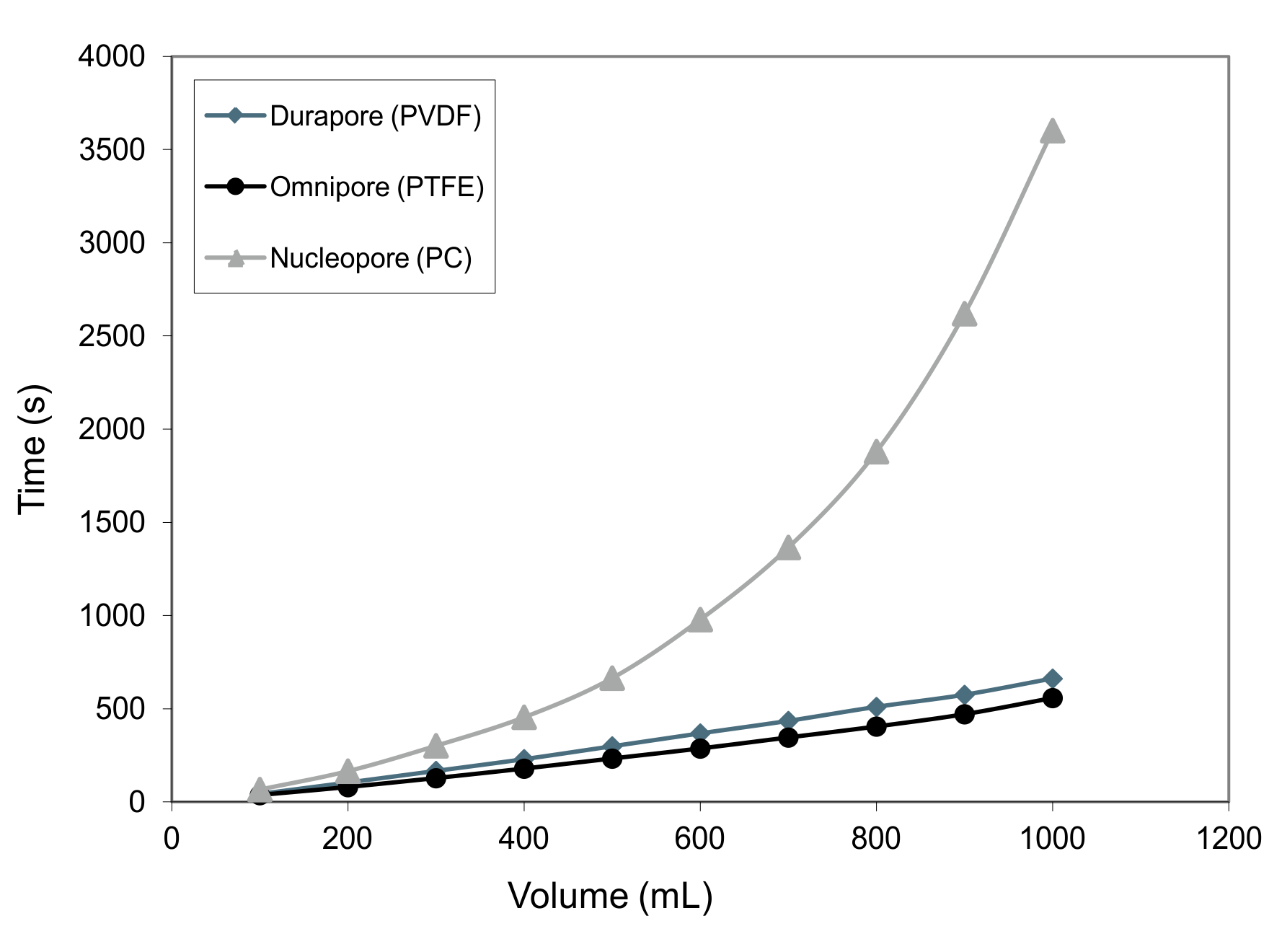

Supplement: S1 Fig — Three filters of 0.2 μm pore-size and 47 mm of diameter made of different materials were used. Aliquots of 1 L of a seawater sample collected on 08/11/10 in Mediterranean Sea (41 39.7 N 02 54.6) were filtered onto each filter. (TIF) [file pone.0133207.s001.tif]

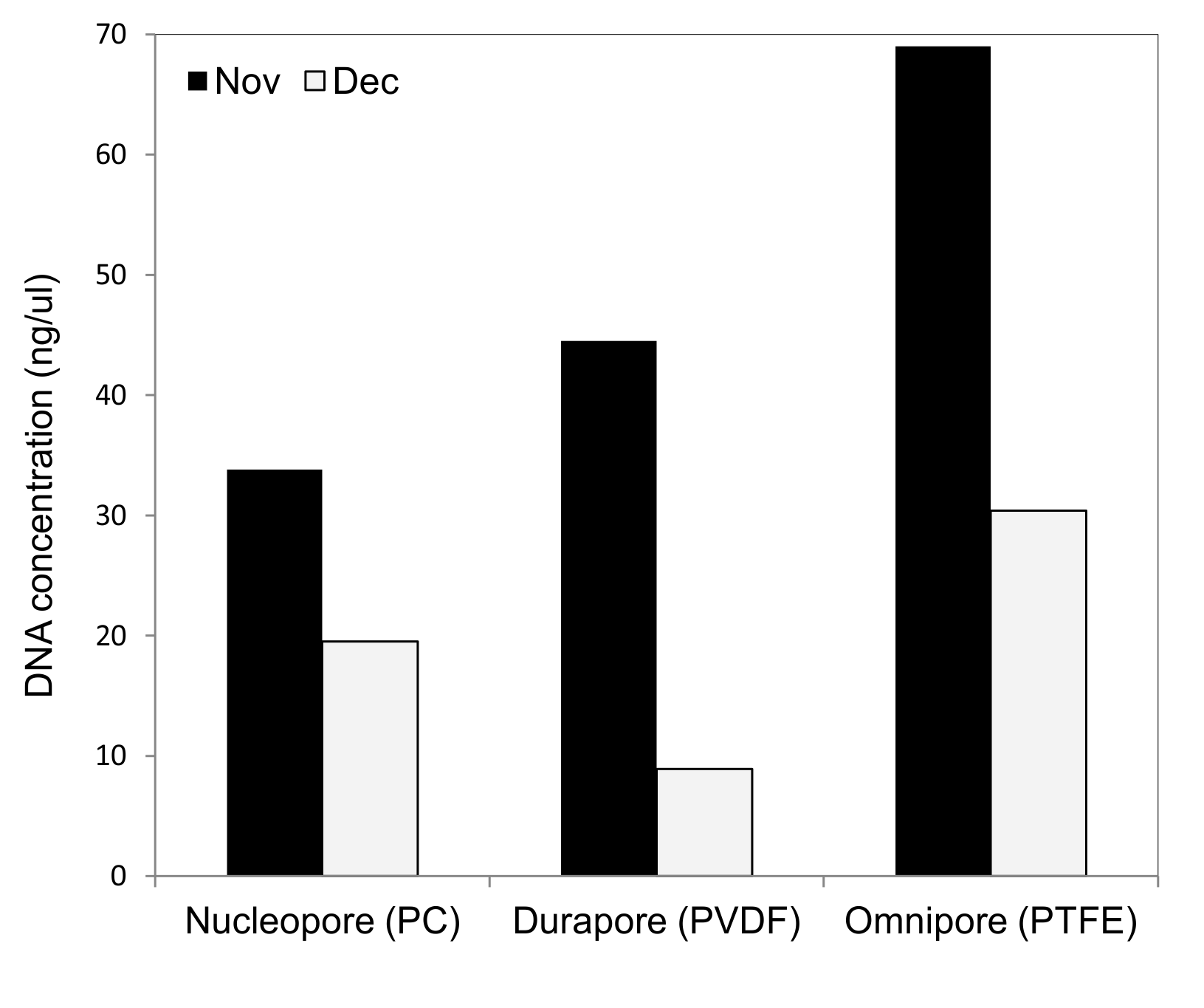

Supplement: S2 Fig — Two water samples of Mediterranean Sea (41 39.7 N 02 54.6) were collected on 11/11/2010 (Nov) and 16/12/2010 (Dec), respectively, using three filters of 0.2 μm pore size and 47 mm of diameter made of different materials. 1 L of water was filtered through each filter. (TIF) [file pone.0133207.s002.tif]

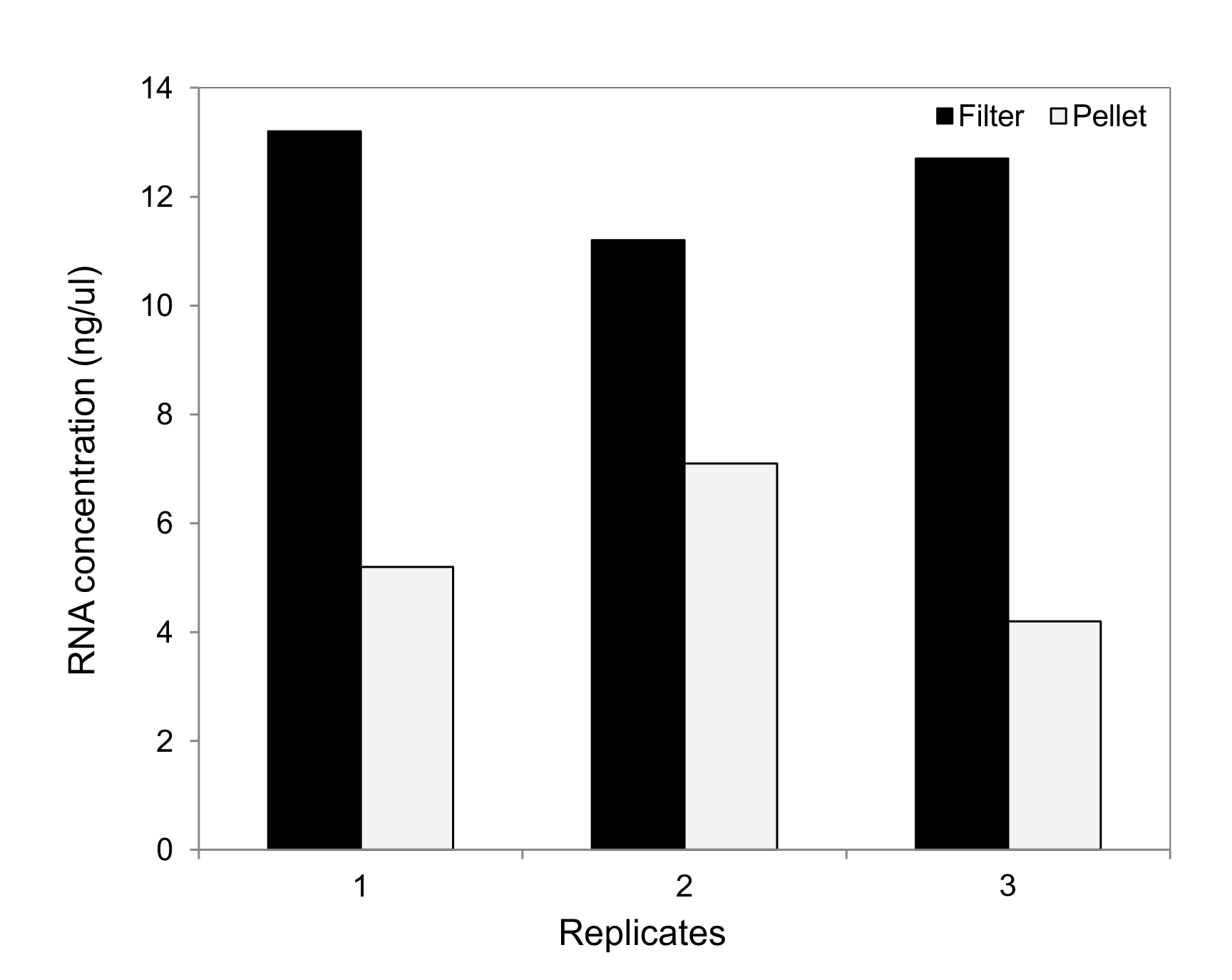

Supplement: S3 Fig — RNA isolated from both the filter and the pellet resulting from centrifugation of RNAlater where the filter was immersed. Three replicates of 1 L of Mediterranean Sea (41 39.7 N 02 54.6) water filtered onto Ominipore (PTFE) filters are shown. RNA concentration obtained from Omnipore (PTFE) filter is between two and three fold that obtained from the pellet. (TIF) [file pone.0133207.s003.tif]

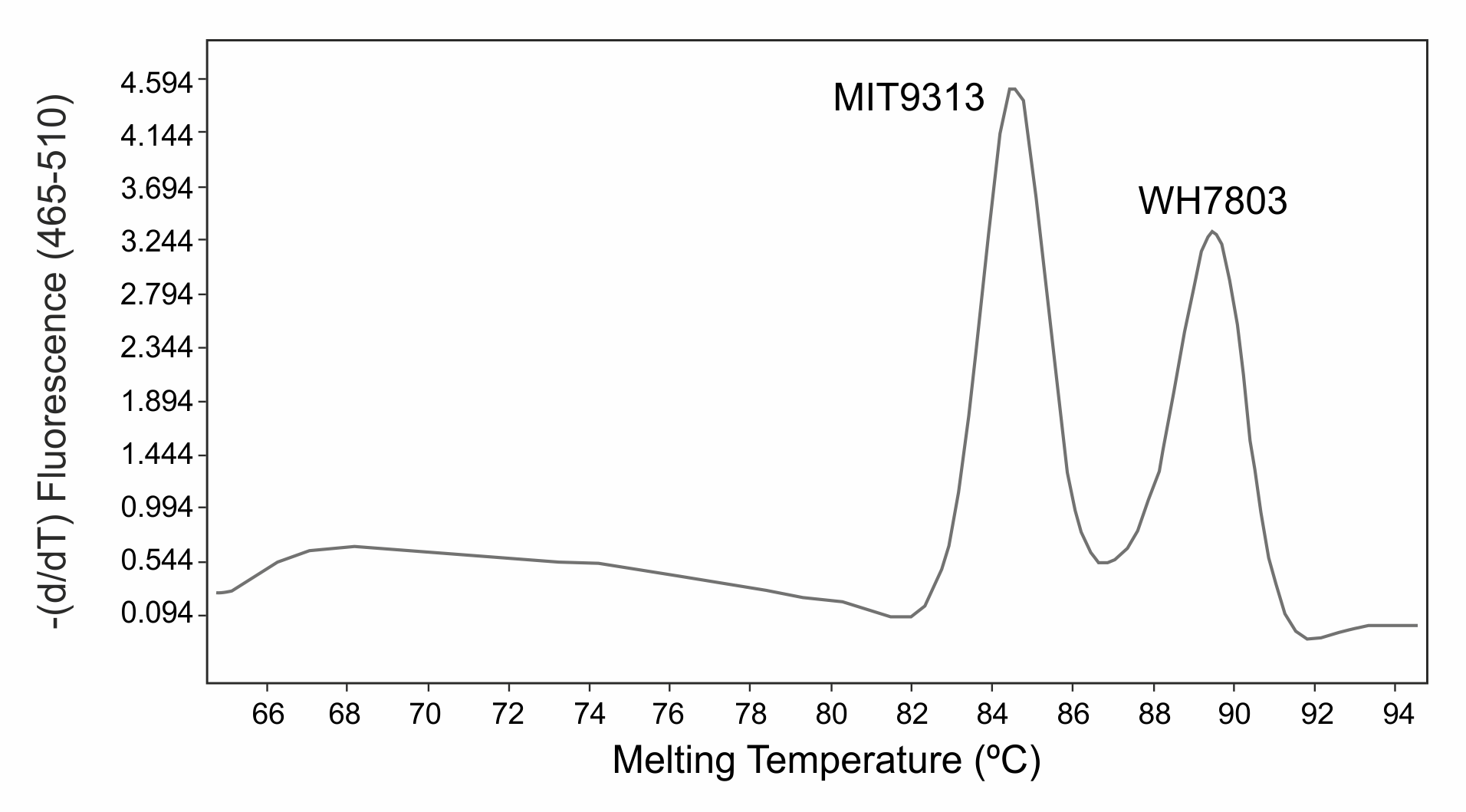

Supplement: S4 Fig — The graphs shows melting temperature curves of amplicons using rbcL LL primers. Melting peaks for Prochlorococcus str. MIT9313 and Synechococcus str. WH7803 in the same sample are shown (TIF) [file pone.0133207.s004.tif]
