# Supplementary material for: Clade-Specific Quantitative Analysis of Photosynthetic Gene Expression in Prochlorococcus
Source: PLoS One. 2015 Aug 5;10(8):e0133207. doi: 10.1371/journal.pone.0133207 (PMC4526520; doi:10.1371/journal.pone.0133207)
Supplement: S1 Table — Prochlorococcus and Synechococcus strains used to design specific primers for Prochlorococcus of the selected genes by sequences alignments using Software Geneious. (DOCX) [file pone.0133207.s005.docx]

| **S1 Table. Model strain list.** *Prochlorococcus* and *Synechococcus* strains used to design specific primers for *Prochlorococcus* of the selected genes by sequences alignments using Software *Geneious*. | | |
| --- | --- | --- |
| **Strain** | **Species/ Ecotype** | **GenBank accession ID** |
| AS9601 | *Prochlorococcus*: High-light | [CP000551](http://www.ncbi.nlm.nih.gov/nucleotide/CP000551) |
| MED4 | *Prochlorococcus*: High-light | [BX548174](http://www.ncbi.nlm.nih.gov/nucleotide/BX548174) |
| MIT9301 | *Prochlorococcus*: High-light | [CP000576](http://www.ncbi.nlm.nih.gov/nucleotide/CP000576) |
| MIT9312 | *Prochlorococcus*: High-light | [CP000111](http://www.ncbi.nlm.nih.gov/nucleotide/CP000111) |
| MIT9515 | *Prochlorococcus*: High-light | [CP000552](http://www.ncbi.nlm.nih.gov/nucleotide/CP000552) |
| MIT9215 | *Prochlorococcus*: High-light | [CP000825](http://www.ncbi.nlm.nih.gov/nucleotide/CP000825) |
| MIT9313 | *Prochlorococcus*: Low-light | [BX548175](http://www.ncbi.nlm.nih.gov/nucleotide/BX548175) |
| MIT9303 | *Prochlorococcus*: Low-light | [CP000554](http://www.ncbi.nlm.nih.gov/nucleotide/CP000554) |
| MIT9211 | *Prochlorococcus*: Low-light | [CP000878](http://www.ncbi.nlm.nih.gov/nucleotide/CP000878) |
| NATL1A | *Prochlorococcus*: Low-light | [CP000553](http://www.ncbi.nlm.nih.gov/nucleotide/CP000553) |
| NATL2A | *Prochlorococcus*: Low-light | [CP000095](http://www.ncbi.nlm.nih.gov/nucleotide/CP000095) |
| SS120 | *Prochlorococcus*: Low-light | [AE017126](http://www.ncbi.nlm.nih.gov/nucleotide/AE017126) |
| CC9311 | *Synechococcus* | [CP000435](http://www.ncbi.nlm.nih.gov/nucleotide/CP000435) |
| CC9902 | *Synechococcus* | [CP000097](http://www.ncbi.nlm.nih.gov/nucleotide/CP000097) |
| RCC307 | *Synechococcus* | [CT978603](http://www.ncbi.nlm.nih.gov/nucleotide/CT978603) |
| CC9605 | *Synechococcus* | [CP000110](http://www.ncbi.nlm.nih.gov/nucleotide/CP000110) |
| WH7803 | *Synechococcus* | [CT971583](http://www.ncbi.nlm.nih.gov/nucleotide/CT971583) |
