# Supplementary material for: Clade-Specific Quantitative Analysis of Photosynthetic Gene Expression in Prochlorococcus
Source: PLoS One. 2015 Aug 5;10(8):e0133207. doi: 10.1371/journal.pone.0133207 (PMC4526520; doi:10.1371/journal.pone.0133207)
Supplement: S2 Table — (DOCX) [file pone.0133207.s006.docx]

| **S2 Table. Coordinates and collection data of field samples collected during Malaspina cruise.** | | | | | | |
| --- | --- | --- | --- | --- | --- | --- |
| **Station Number** | **Longitude (DD)** | **Latitude (DD)** | **Collection Date (dd-mm-yy)** | **DCM Depth (m)** | **Collection local time (3 m depth sample)** | **Collection local time (DCM & DCM+40 samples)** |
| 1 | -9.5 | 35.2 | 16-12-10 | 130 | 9:44 | 11:43 |
| 2 | -26.0 | 9.6 | 28-12-10 | 70 | 8:50 | 11:11 |
| 3 | -26.0 | 5.0 | 30-12-10 | 120 | 8:16 | 10:35 |
| 4 | -26.0 | 0.3 | 01-01-11 | 62 | 8:30 | 11:05 |
| 5 | -28.2 | -4.8 | 03-01-11 | 110 | 9:30 | 12:00 |
| 6 | -30.2 | -9.1 | 05-01-11 | 150 | 9:30 | 11:50 |
| 7 | -32.4 | -13.7 | 07-01-11 | 150 | 9:23 | 12:09 |
| 8 | -34.7 | -18.4 | 09-01-11 | 130 | 9:20 | 11:55 |
| 9 | -37.0 | -23.0 | 11-01-11 | 150 | 9:32 | 12:10 |
| 10 | -36.2 | -24.3 | 19-01-11 | 119 | 8:00 | 11:00 |
| 11 | -33.1 | -24.9 | 20-01-11 | 120 | 8:40 | 11:22 |
| 12 | -30.1 | -25.4 | 21-01-11 | 120 | 9:00 | 10:30 |
| 13 | -21.4 | -26.9 | 24-01-11 | 125 | 8:45 | 11:15 |
| 14 | -14.8 | -28.1 | 26-01-11 | 150 | 8:40 | 11:04 |
| 15 | -5.4 | -29.7 | 29-01-11 | 110 | 8:45 | 11:27 |
| 16 | 3.7 | -31.3 | 01-02-11 | 70 | 8:00 | 12:00 |
| 17 | 6.8 | -31.8 | 02-02-11 | 85 | 9:00 | 11:00 |
| 18 | 9.4 | -32.1 | 03-02-11 | 72 | 8:45 | 11:45 |
| 19 | 12.7 | -32.8 | 04-02-11 | 48 | 8:45 | 11:45 |
| 20 | 15.5 | -33.3 | 05-02-11 | 44 | 8:00 | 10:00 |
| 21 | 27.5 | -34.8 | 14-02-11 | 96 | 8:30 | 11:52 |
| 22 | 33.7 | -34.2 | 16-02-11 | 75 | 7:52 | 9:40 |
| 23 | 39.9 | -33.5 | 18-02-11 | 125 | 8:26 | 9:42 |
| 24 | 43.2 | -33.2 | 19-02-11 | 110 | 7:46 | 9:57 |
| 25 | 61.5 | -30.1 | 24-02-11 | 130 | 8:15 | 10:22 |
| 26 | 63.2 | -28.0 | 25-02-11 | 112 | 8:38 | 10:38 |
| 27 | 69.4 | -29.4 | 27-02-11 | 130 | 9:11 | 11:04 |
| 28 | 76.1 | -29.9 | 01-03-11 | 140 | 8:12 | 10:48 |
| 29 | 82.6 | -29.8 | 03-03-11 | 135 | 8:12 | 10:08 |
| 30 | 89.5 | -29.7 | 05-03-11 | 120 | 8:05 | 10:12 |
| 31 | 96.4 | -29.6 | 07-03-11 | 114 | 8:09 | 10:41 |
| 32 | 103.3 | -30.3 | 09-03-11 | 100 | 8:13 | 10:29 |
| 33 | 110.2 | -31.2 | 11-03-11 | 90 | 8:16 | 10:24 |
| 34 | 135.2 | -39.2 | 24-03-11 | 70 | 8:30 | 11:02 |
| 35 | -178.2 | -23.4 | 20-04-11 | 110 | 8:40 | 11:22 |
| 36 | -176.9 | -20.6 | 21-04-11 | 110 | − | 11:21 |
| 37 | -174.5 | -15.9 | 23-04-11 | 105 | 8:05 | 9:41 |
| 38 | -172.3 | -9.5 | 26-04-11 | 115 | 7:20 | 10:20 |
| 39 | -168.4 | -1.3 | 30-04-11 | 65 | 7:20 | 10:32 |
| 40 | -165.8 | 3.8 | 02-05-11 | 80 | 7:15 | 9:43 |
| 41 | -164.4 | 7.0 | 03-05-11 | 80 | 8:50 | 8:12 |
| 42 | -160.9 | 15.0 | 06-05-11 | 140 | 10:05 | 9:51 |
| 43 | -150.4 | 21.1 | 16-05-11 | 105 | 7:17 | 10:29 |
| 44 | -145.2 | 20.3 | 18-05-11 | − | 7:05 | − |
| 45 | -139.0 | 19.3 | 20-05-11 | 130 | 7:19 | 10:42 |
| 46 | -133.3 | 18.1 | 22-05-11 | 125 | 7:16 | 10:32 |
| 47 | -127.6 | 16.6 | 24-05-11 | 100 | 9:25 | 10:04 |
| 48 | -122.0 | 15.3 | 26-05-11 | 137 | 7:22 | 10:26 |
| 49 | -115.8 | 13.8 | 28-05-11 | 90 | 7:20 | 10:05 |
| 50 | -110.4 | 12.5 | 30-05-11 | 125 | 7:19 | 11:36 |
| 51 | -102.4 | 10.8 | 02-06-11 | 37 | 7:33 | 9:52 |
| 52 | -96.3 | 9.4 | 04-06-11 | 19 | 7:20 | 10:09 |
| 53 | -93.1 | 8.8 | 05-06-11 | 24 | 7:18 | 11:20 |
| 54 | -87.9 | 7.2 | 07-06-11 | 20 | 8:34 | 10:11 |
| 55 | -69.3 | 15.1 | 22-06-11 | 95 | 7:08 | 10:52 |
| 56 | -55.2 | 19.0 | 27-06-11 | 140 | 7:20 | 10:58 |
| 57 | -47.8 | 21.7 | 30-06-11 | 120 | 7:46 | 11:04 |
| 58 | -41.9 | 23.7 | 02-07-11 | 130 | 7:18 | 10:04 |
| 59 | -35.3 | 26.1 | 04-07-11 | 150 | 7:15 | 10:07 |
| 60 | -29.7 | 28.0 | 06-07-11 | 140 | 7:13 | 10:13 |
| 61 | -23.7 | 30.0 | 08-07-11 | 100 | 7:20 | 10:23 |
| 62 | -17.3 | 32.1 | 10-07-11 | 110 | 7:14 | 10:20 |
