# Supplementary material for: Clade-Specific Quantitative Analysis of Photosynthetic Gene Expression in Prochlorococcus
Source: PLoS One. 2015 Aug 5;10(8):e0133207. doi: 10.1371/journal.pone.0133207 (PMC4526520; doi:10.1371/journal.pone.0133207)
Supplement: S3 Table — Specificity of the designed primers checked by qRT-PCR using pure cultures of some Prochlorococcus and Synechococcus strains at equal cell concentrations and verification of the method validity in natural communities*. (DOCX) [file pone.0133207.s007.docx]

| **S3 Table. Primer specificity.** Specificity of the designed primers checked by qRT-PCR using pure cultures of some *Prochlorococcus* and *Synechococcus* strains at equal cell concentrations and verification of the method validity in natural communities*. | | | | | | | | | | | | | | | | | | | | | |  |
| --- | --- | --- | --- | --- | --- | --- | --- | --- | --- | --- | --- | --- | --- | --- | --- | --- | --- | --- | --- | --- | --- | --- |
|  | | | **High-light primers** | | | | | | | | |  | **Low -light primers** | | | | | | | | | |
|  |  |  | *rnp*B | | | *rbc*L | | | *psb*A | | |  | *rnp*B | | | *rbc*L | | | *psb*A | | | |
|  |  |  | Cp | Tm_1_ | Tm_2_ | Cp | Tm_1_ | Tm_2_ | Cp | Tm_1_ | Tm_2_ |  | Cp | Tm_1_ | Tm_2_ | Cp | Tm_1_ | Tm_2_ | Cp | Tm_1_ | Tm_2_ | |
| **Axenic cultures** | HL *Prochlorococcus* | MED4 | 17.38 | 81.14 | n/d | 20.04 | 84.96 | n/d | 15.72 | 81.75 | n/d |  | 34.09 | 77.00 | n/d | 37.80 | 78.00 | n/d | 31.89 | 87.11 | 84.32 | |
|  |  | MIT9515 | 14.86 | 81.05 | n/d | 18.41 | 84.53 | n/d | 13.18 | 81.60 | n/d |  | 33.49 | 87.92 | 84.28 | 31.52 | 84.73 | n/d | 33.14 | 87.32 | 83.90 | |
|  |  | EQPAC1-C | 19.26 | 80.52 | n/d | 18.90 | 84.82 | n/d | 17.09 | 81.58 | n/d |  | 30.85 | 84.88 | 87.87 | 37.93 | 81.14 | 78.90 | 34.95 | 83.43 | 77.54 | |
|  |  |  |  |  |  |  |  |  |  |  |  |  |  |  |  |  |  |  |  |  |  | |
|  | LL *Prochlorococcus* | MIT9313 | 32.75 | 80.96 | n/d | 33.45 | 87.36 | 81.59 | 27.98 | 87.99 | n/d |  | 15.40 | 87.98 | n/d | 18.29 | 84.92 | n/d | 14.08 | 84.69 | n/d | |
|  |  | NATL2A | 30.11 | 80.95 | n/d | 31.37 | 84.97 | n/d | 29.67 | 81.59 | n/d |  | 18.09 | 86.90 | n/d | 21.02 | 81.30 | n/d | 14.14 | 84.92 | n/d | |
|  |  |  |  |  |  |  |  |  |  |  |  |  |  |  |  |  |  |  |  |  |  | |
|  | *Synechococcus* | WH7803 | 32.81 | 81.05 | n/d | 33.14 | 84.41 | 81.90 | 28.66 | 81.35 | n/d |  | 17.83 | 88.01 | n/d | 28.27 | 89.62***** | n/d | 28.26 | 84.53 | 78.92 | |
|  |  |  |  |  |  |  |  |  |  |  |  |  |  |  |  |  |  |  |  |  |  | |
|  | MIT9313 + WH7803  (equal cell concentration) | | 33.48 | 81.29 | n/d | 33.52 | 87.47 | 87.57 | 29.91 | 81.43 | n/d |  | 15.49 | 88.03 | n/d | 23.14 | 84.60 | 89.38***** | 16.25 | 86.82 | n/d | |
|  |  | |  |  |  |  |  |  |  |  |  |  |  |  |  |  |  |  |  |  |  | |
| **Field samples** | Atlantic | 3 m | 21.08 | 81.31 | n/d | 21.49 | 84.27 | n/d | 18.82 | 81.99 | n/d |  | 26.56 | 88.80 | n/d | 34.02 | 85.75 | 88.88***** | 28.67 | 87.85 | n/d | |
|  |  | DCM | 21.21 | 81.08 | n/d | 21.59 | 83.77 | n/d | 17.87 | 81.42 | n/d |  | 20.87 | 85.31 | n/d | 23.83 | 82.92 | n/d | 17.72 | 84.1 | n/d | |
|  |  | DCM+40 | 27.84 | 81.03 | n/d | 29.00 | 84.08 | n/d | 25.26 | 81.67 | n/d |  | 24.12 | 84.95 | 87.83 | 27.44 | 84.61 | n/d | 22.56 | 85.45 | n/d | |
|  |  |  |  |  |  |  |  |  |  |  |  |  |  |  |  |  |  |  |  |  |  | |
|  | Indian | 3 m | 20.76 | 81.29 | n/d | 21.42 | 83.78 | n/d | 15.87 | 81.50 | n/d |  | 29.06 | 88.98 | 85.46 | 31.97 | 89.38***** | 86.54 | 25.73 | 86.87 | n/d | |
|  |  | DCM | 23.38 | 81.34 | n/d | 25.78 | 84.75 | n/d | 21.69 | 81.87 | n/d |  | 18.46 | 85.97 | n/d | 22.42 | 83.09 | n/d | 16.41 | 84.32 | n/d | |
|  |  | DCM+40 | 30.39 | 81.54 | n/d | 31.78 | 85.03 | n/d | 25.56 | 82.22 | n/d |  | 19.35 | 84.99 | n/d | 24.99 | 83.61 | n/d | 18.56 | 84.57 | n/d | |
|  |  |  |  |  |  |  |  |  |  |  |  |  |  |  |  |  |  |  |  |  |  | |
|  | Pacific 1 | 3 m | 20.99 | 81.09 | n/d | 20.05 | 83.09 | n/d | 16.78 | 81.76 | n/d |  | 26.06 | 86.63 | 89.15 | 27.94 | 83.11 | 87.96 | 22.96 | 84.30 | 87.59 | |
|  |  | DCM | 22.59 | 80.77 | n/d | 25.83 | 83.31 | n/d | 21.59 | 81.68 | n/d |  | 18.55 | 85.74 | n/d | 23.74 | 83.21 | n/d | 17.80 | 84.35 | n/d | |
|  |  | DCM+40 | 26.05 | 81.30 | n/d | 29.04 | 83.71 | n/d | 23.44 | 81.63 | n/d |  | 19.15 | 85.06 | n/d | 24.07 | 83.45 | n/d | 18.55 | 84.49 | n/d | |
|  |  |  |  |  |  |  |  |  |  |  |  |  |  |  |  |  |  |  |  |  |  | |
|  | Pacific 2 | 3 m | 18.88 | 80.38 | n/d | 17.67 | 83.69 | n/d | 15.04 | 82.00 | n/d |  | 22.05 | 88.90 | n/d | 32.02 | 88.52***** | 82.93 | 24.98 | 88.03 | 84.22 | |
|  |  | DCM | 19.59 | 80.09 | n/d | 18.97 | 83.69 | n/d | 14.29 | 81.99 | n/d |  | 20.50 | 89.07 | 85.36 | 34.18 | 82.64 | 86.04 | 29.12 | 84.15 | n/d | |
|  |  | DCM+40 | 23.75 | 80.48 | n/d | 26.30 | 83.99 | n/d | 20.28 | 82.00 | n/d |  | 18.03 | 87.49 | n/d | 24.80 | 86.09 | n/d | 16.49 | 87.30 | n/d | |
|  |  |  |  |  |  |  |  |  |  |  |  |  |  |  |  |  |  |  |  |  |  | |
| * Second derivate maximum of the amplification curves (Cp; nº of cycle) are shown as calculated for 1.1 ng/ml of cDNA per qPCR reaction by averaging of triplicates. Cp values were calculated by the Second Derivate Maximum method. Melting temperatures (Tm; ºC) are shown for the main PCR product (Tm_1_) and the minor one (Tm_2_), in the case that two different products (i.e. different sequences) are amplified. When Tm > 88ºC for *rbc*L LL primers the PCR product is considered to belong to *Synechococcus* (red asterisk ***** ). n/d means value not detected. | | | | | | | | | | | | | | | | | | | | | | |
