# Supplementary material for: Clade-Specific Quantitative Analysis of Photosynthetic Gene Expression in Prochlorococcus
Source: PLoS One. 2015 Aug 5;10(8):e0133207. doi: 10.1371/journal.pone.0133207 (PMC4526520; doi:10.1371/journal.pone.0133207)
Supplement: S4 Table — Sequencing of single amplicons from samples collected at three different depths and three sampled station during Malaspina circumnavigation. (DOCX) [file pone.0133207.s008.docx]

| **S4 Table. Amplicon sequences.** Sequencing of single amplicons from samples collected at three different depths and three sampled station during Malaspina circumnavigation. | | | | | | | | | | | | | | | | |
| --- | --- | --- | --- | --- | --- | --- | --- | --- | --- | --- | --- | --- | --- | --- | --- | --- |
| **Station^a^** | | **Depth** | **Amplicon^b^** | **rnpB_HL** | **Ident(%)^c^** | **rbcL_HL** | **Ident (%) ^c^** | **psbA_HL** | **Ident (%) ^c^** | **rnpB_LL** | **Ident(%) ^c^** | **rbcL_LL** | **Ident(%) ^c^** | **psbA_LL** | | **Ident. (%) ^c^** |
| **Atlantic** | | 3 m | 1 | MIT9312 (HL) | 100 | MIT9515 (HL) | 100 | MIT9215, MIT9301, MIT9302 (HL) | 99 | No similarity found |  | *Syn.* CB0103, CB0102 | 93 | *Syn*. RSS9907 | | 95 |
|  |  |  | 2 | MIT9312 (HL) | 100 | MIT9515 (HL) | 100 | MIT9215, MIT9301, MIT9302 (HL) | 96 | MIT9313, MIT9211, MIT9303 (LL); *Syn*.: WH7803, CC9311, CC9605, CC9902, WH8102, PCC7001 | 87* | *Syn.* CB0205 | 89 | *Syn*. RSS9907 | | 94 |
|  |  | DCM | 1 | MIT9312 (HL) | 100 | MIT9515 (HL) | 98 | MIT9215, MIT9301, MIT9302 (HL) | 98 | NATL2A (LL) | 95 | SS120 (LL) | 89 | NATL2A, NATL1A (LL) | | 98 |
|  |  |  | 2 | MIT9312 (HL) | 100 | MIT9515 (HL) | 100 | MIT9215, MIT9301, MIT9302 (HL) | 98 | NATL2A (LL) | 94 | MIT9313 (LL) | 89 | NATL2A, NATL1A (LL) | | 98 |
|  |  | DCM+40 | 1 | MIT9312 (HL) | 98 | MIT9515 (HL) | 100 | MIT9215, MIT9301, MIT9302 (HL) | 96 | MIT9303 (LL) | 85* | SS120 (LL) | 88 | MIT9211 (LL) | | 88 |
|  |  |  | 2 | MIT9312 (HL) | 100 | AS9601 (HL) | 98 | MIT9215, MIT9301, MIT9302 (HL) | 98 | MIT9303 (LL) | 85 | MIT9313 (LL) | 90 | MIT9211 (LL) | | 87 |
|  | |  |  |  |  |  |  |  |  |  |  |  |  |  | |  |
| **Indian** | | 3 m | 1 | MIT9312 (HL) | 98 | MIT9215, MIT9515 (HL) | 98 | MED4, AS9601, MIT9312, MIT9116 (HL) | 98 | NATL2A. NATL1A, PAC1B, PAC1A (LL) | 96* | NATL2A, NATL1A (LL) | 85 | *Syn*. WH8109, RSS9907 | | 97 |
|  |  |  | 2 | MIT9312 (HL) | 100 | MED4 (HL) | 98 | MED4, MIT9116 (HL) | 94 | MIT9313, MIT9211, MIT9303 (LL); *Syn*.: WH7803, CC9311, CC9605, CC9902, WH8102, PCC7001 | 92 | *Syn*. WH7803, WH8102, WH8108, WH8104, WH8103, WH8008, WH8006, WH7805 | 95* | *Syn*. WH8109, RSS9907 | | 96 |
|  |  | DCM | 1 | MIT9212 (HL) | 96 | MED4(HL) | 97 | MED4, MIT9312, MIT9116 (HL) | 98 | NATL2A. NATL1A, PAC1B, PAC1A (LL) | 92-98 | NATL2A, NATL1A (LL) | 91 | NATL2A, NATL1A (LL) | | 96 |
|  |  |  | 2 | MIT9312 (HL) | 94 | MIT9515 (HL) | 95 | MED4, MIT9312, MIT9116 (HL) | 97 | NATL2A. NATL1A, PAC1B, PAC1A (LL) | 98 | NATL2A, NATL1A (LL) | 92 | NATL2A, NATL1A (LL) | | 97 |
|  |  | DCM+40 | 1 | No amplification |  | MIT9515 (HL) | 90 | MIT9215, MIT9301, MIT9302 (HL) | 91 | NATL2A. NATL1A, PAC1B, PAC1A (LL) | 96 | MIT9313 (LL) | 88 | NATL2A, NATL1A (LL) | | 96 |
|  |  |  | 2 | No amplification |  | MIT9515 (HL) | 98* | MIT9215, MIT9301, MIT9302 (HL) | 92 | NATL2A. NATL1A, PAC1B, PAC1A (LL) | 95 | MIT9313 (LL) | 91 | NATL2A, NATL1A (LL) | | 94 |
|  | |  |  |  |  |  |  |  |  |  |  |  |  |  | |  |
| **Pacific1** | | 3 m | 1 | MIT9312 (HL) | 100 | MIT9515 (HL) | 98 | MIT9215, MIT9301, MIT9302 (HL) | 98 | NATL2A. NATL1A, PAC1B, PAC1A (LL) | 84 | NATL2A, NATL1A (LL) | 95 | NATL2A, NATL1A (LL) | | 96 |
|  |  |  | 2 | MIT9312 (HL) | 100 | MIT9515 (HL) | 98 | MIT9215, MIT9301, MIT9302 (HL) | 97 | NATL2A. NATL1A, PAC1B, PAC1A (LL) | 94 | NATL2A, NATL1A (LL) | 92 | NATL2A, NATL1A (LL) | | 92 |
|  |  | DCM | 1 | MIT9312 (HL) | 100 | MIT9515 (HL) | 98 | MIT9215, MIT9301, MIT9302 (HL) | 99 | NATL2A. NATL1A, PAC1B, PAC1A (LL) | 93 | NATL2A, NATL1A (LL) | 92 | NATL2A, NATL1A (LL) | | 96 |
|  |  |  | 2 | MIT9312 (HL) | 100 | MIT9515 (HL) | 98 | MIT9215, MIT9301, MIT9302 (HL) | 98 | NATL2A. NATL1A, PAC1B, PAC1A (LL) | 94 | NATL2A, NATL1A (LL) | 93 | NATL2A, NATL1A (LL) | | 98 |
|  |  | DCM+40 | 1 | MIT9312 (HL) | 100 | MIT9515 (HL) | 97 | MIT9215, MIT9301, MIT9302 (HL) | 94 | NATL2A. NATL1A, PAC1B, PAC1A (LL) | 92 | MIT9313 (LL) | 91 | NATL2A, NATL1A (LL) | | 93 |
|  |  |  | 2 | MIT9312 (HL) | 100 | MIT9515 (HL) | 94 | MIT9215, MIT9301, MIT9302 (HL) | 97 | NATL2A. NATL1A, PAC1B, PAC1A (LL) | 95 | MIT9313 (LL) | 89 | NATL2A, NATL1A (LL) | | 94 |
|  | |  |  |  |  |  |  |  |  |  |  |  |  |  | |  |
| **Pacific2** | | 3 m | 1 | MIT9301, AS9601, MIT9312 (HL) | 99-100 | AS9601 (HL) | 99 | MIT9215, MIT9301, MIT9302 (HL) | 98 | *Syn*: CC9605, CC9902 | 99 | NATL2A, NATL1A (LL) | 93 | NATL2A, NATL1A (LL) | | 97 |
|  |  | DCM | 1 | AS9601, TATL2 (HL) | 93 | MIT9301 (HL) | 98 | MIT9301, MIT9302(HL) | 99 | NATL2A. NATL1A, PAC1B, PAC1A (LL) | 95 | MIT9313, MIT9303 (LL) | 89 | NATL2A, NATL1A (LL) | | 97 |
|  |  | DCM+40 | 1 | TAK9803 (HL) | 99 | MIT9301, MIT9515, AS9601 (HL) | 94 | MIT9215, MIT9301, MIT9312, MIT9302 (HL) | 97 | MIT9211, MIT9303 (LL), SYN.WH8102 | 85 | MIT9313 (LL) | 87 | NATL2A, NATL1A (LL) | | 89 |
|  | ^a^ Station data in are shown in Table 2. ^b^ Two qRT-PCR products were sequenced for each sample from Atlantic, Indian and Pacific1 stations, and only one for Pacific2 samples. ^c^ Results of maximum score from BLAST are shown. All sequences showed a coverage higher than 70% except the marked with “ * “, that showed a coverage between 58-69%. ^d^ *Synechococcus* strains are indicated as “*Syn*”. | | | | | | | | | | | | | |  |  |
