# Supplementary material for: Clade-Specific Quantitative Analysis of Photosynthetic Gene Expression in Prochlorococcus
Source: PLoS One. 2015 Aug 5;10(8):e0133207. doi: 10.1371/journal.pone.0133207 (PMC4526520; doi:10.1371/journal.pone.0133207)
Supplement: S5 Table — Paired-sample Wilcoxon signed rank tests achieved with control- treatment pairs of cell concentration measured. (DOCX) [file pone.0133207.s009.docx]

| **S5 Table. Cell concentration variability.** P[aired-sample Wilcoxon signed rank](http://www.originlab.com/index.aspx?go=Products/Origin/Statistics/NonparametricTests&pid=1084)tests achieved with control- treatment pairs of cell concentration measured. | | | | | | | |
| --- | --- | --- | --- | --- | --- | --- | --- |
|  | |  | | **Cell concentration** [**Paired-Sample Wilcoxon Signed Rank Test**](http://www.originlab.com/index.aspx?go=Products/Origin/Statistics/NonparametricTests&pid=1084) | | | |
| **Strain** | | **Treatment** | | **N** | | **Sig.** | |
| MED4 | | PAHs | | 6 | | 0.753 | |
|  | | OClP | | 6 | | 0.917 | |
|  | |  | |  | |  | |
| MIT9313 | | PAHs | | 6 | | 0.917 | |
|  | | OClP | | 6 | | 0.345 | |
| **S5 Table. Cell concentration variability.** P[aired-sample Wilcoxon signed rank](http://www.originlab.com/index.aspx?go=Products/Origin/Statistics/NonparametricTests&pid=1084)tests achieved with control- treatment pairs of cell concentration measured. | | | | | | | |
|  | |  | | **Cell concentration** [**Paired-Sample Wilcoxon Signed Rank Test**](http://www.originlab.com/index.aspx?go=Products/Origin/Statistics/NonparametricTests&pid=1084) | | | |
| **Strain** | | **Treatment** | | **N** | | **Sig.** | |
| MED4 | | PAHs | | 6 | | 0.753 | |
|  | | OClP | | 6 | | 0.917 | |
|  | |  | |  | |  | |
| MIT9313 | | PAHs | | 6 | | 0.917 | |
|  | | OClP | | 6 | | 0.345 | |
