# Supplementary material for: Clade-Specific Quantitative Analysis of Photosynthetic Gene Expression in Prochlorococcus
Source: PLoS One. 2015 Aug 5;10(8):e0133207. doi: 10.1371/journal.pone.0133207 (PMC4526520; doi:10.1371/journal.pone.0133207)
Supplement: S6 Table — Results from comparing means test achieved with control- treatment pairs of chlorophyll a fluorescence. (DOCX) [file pone.0133207.s010.docx]

| **S6 Table. Changes in chlorophyll a concentrations.** Results from comparing means test achieved with control- treatment pairs of chlorophyll a fluorescence. | | | | |
| --- | --- | --- | --- | --- |
|  |  | | **Fluorescence Paired T-test** | |
|  | |  | df | Sig. (2-tailed) |
| All experiments | | All | 20 | 0.679 |
| Strain | | MED4 | 10 | 0.341 |
|  |  | MIT9313 | 10 | 0.765 |
| Treatment | | PAHs | 8 | 0.888 |
|  |  | OClP | 11 | 0.508 |
| Exposure time | | 0.5h | 9 | 0.898 |
|  |  | 24h | 11 | 0.458 |
