# Supplementary material for: Clade-Specific Quantitative Analysis of Photosynthetic Gene Expression in Prochlorococcus
Source: PLoS One. 2015 Aug 5;10(8):e0133207. doi: 10.1371/journal.pone.0133207 (PMC4526520; doi:10.1371/journal.pone.0133207)
Supplement: S7 Table — Relative changes in DNA abundance for different genes in MIT9313 and MED4 Prochlorococcus cultures when challenged with organic pollutant treatments (PAHs or OClP), evaluated at two different incubation times (0.5 and 24 hours). Results from four different paired T-tests are shown: one of the whole data set (“All samples”) and one of each data subset (“Strain”, “Treatment” and “Incubation time”). (DOCX) [file pone.0133207.s011.docx]

| **S7 Table. Quantitative Genomic changes.** Relative changes in DNA abundance for different genes in MIT9313 and MED4 *Prochlorococcus* cultures when challenged with organic pollutant treatments (PAHs or OClP), evaluated at two different incubation times (0.5 and 24 hours). Results from four different paired T-tests are shown: one of the whole data set (“All samples”) and one of each data subset (“Strain”, “Treatment” and “Incubation time”). | | | | | |
| --- | --- | --- | --- | --- | --- |
|  |  | Average fold change (95% confidence limits)^a^ | | | *n*^b^ |
| **Ratios** |  | ***rbc*L** | ***psb*A** | ***rbc*L/*psb*A** |  |
| All samples |  | 0.97 (0.92- 1.03) | 0.98 (0.91- 1.06) | 0.99 (0.92- 1.07) | 22 |
| Strain | MIT9313 | 0.92 (0.84- 1.01) | 1.00 (0.86- 1.17) | 0.92 (0.80- 1.05) | 10 |
|  | MED4 | 1.03 (0.97- 1.09) | 0.97 (0.90- 1.04) | 1.06 (1.00- 1.13) | 12 |
| Treatment | PAHs | 0.98 (0.91- 1.06) | 1.03(0.93- 1.14) | 0.95 (0.86- 1.06) | 12 |
|  | OClP | 0.96 (0.86- 1.07) | 0.90 (0.83- 0.97)* | 1.07 (0.97- 1.17) | 10 |
| Incubation time | 0.5h | 0.98 (0.88- 1.08) | 1.01 (0.88- 1.16) | 0.97 (0.87- 1.08) | 11 |
|  | 24h | 0.97 (0.90- 1.04) | 0.96 (0.87- 1.06) | 1.01 (0.89- 1.15) | 11 |
| a) Paired T-test, treated vs. untreated, *, p<0.05  b) Total number of treated/untreated pairs of cultures included in each subset | | | |  |  |
